# Red Mud as an Efficient, Stable, and Cost-Free Catalyst for CO_x_-Free Hydrogen Production from Ammonia

**DOI:** 10.1038/srep32279

**Published:** 2016-08-25

**Authors:** Samira Fatma Kurtoğlu, Alper Uzun

**Affiliations:** 1 Department of Chemical and Biological Engineering, Koç University, Rumelifeneri Yolu, Sariyer, 34450, Istanbul, Turkey; 2 Koç University TÜPRAŞ Energy Center (KUTEM), Koç University Rumelifeneri Yolu, Sariyer 34450, Istanbul, Turkey

## Abstract

Red mud, one of the mostly produced industrial wastes, was converted into a catalyst with exceptionally high and stable performance for hydrogen production from ammonia. Results showed that iron species produced after reduction of the HCl digested red mud were converted into ε-Fe_2_N during the induction period of ammonia decomposition reaction at 700 °C. The catalytic performance measurements indicated that the modified red mud catalyst provides a record high hydrogen production rate for a non-noble metal catalyst at this temperature. For instance, stable hydrogen production rates were measured as 72 and 196 mmol H_2_ min^−1^ g_cat_^−1^ for the corresponding space velocities of 72 000 and 240 000 cm^3^ NH_3_ h^−1^ g_cat_^−1^, respectively, at 700 °C. These results offer opportunities to utilize one of the key hazardous industrial wastes as an eco-friendly, efficient, stable, and almost cost-free catalyst for CO_x_-free hydrogen production from ammonia decomposition.

Concerns related to safe storage of hydrogen have triggered extensive research on developing novel technologies^1^,^2^. Some approaches focus on adsorbing hydrogen on porous materials. These approaches have their own limitations^3^. Others consider storing hydrogen chemically in molecules, such as in CH_4_. However, in most of these storage molecules there is also carbon present, producing unwanted CO_x_ upon decomposition. Ammonia, on the other hand, does not include any carbon, thus it offers opportunities for CO_x_-free hydrogen storage with a high storage density (17.7 wt%).

Several Ru-4,5,6,7,8,9, Ni-10,11,12, and Fe-based^13^,^14^,^15^,^16^ catalysts are proposed for the decomposition of ammonia into hydrogen and nitrogen. Among these catalysts, Ru-based catalysts are known as the most active ones^17^. However, they suffer deeply from limited availability and high cost of ruthenium. Ni- and Fe-based counterparts can be alternative to the noble metal catalysts, as they can produce relatively high hydrogen production rates. For instance, an Fe-based catalyst spatially confined within the tubes of a mesoporous carbon support was shown to provide over 90% initial NH_3_ conversion at a space velocity of 120 000 cm^3^ NH_3_ h^−1^ g_cat_^−1^ at 700 °C^13^. However, such non-noble metal catalysts require elevated temperatures to be able to reach high hydrogen production rates and at these high temperatures they tend to sinter and deactivate quickly, as in the case of this mesoporous carbon supported iron catalyst^13^. Thus, there is a big need for a cheap catalyst with high and stable performance for producing hydrogen from ammonia. Here, the focus is on an industrial waste, red mud (RM), as an environmentally friendly and almost cost-free catalyst.

RM, a by-product of aluminum production process, is formed between 1.9 to 3.6 tons^18^ per each ton of aluminum manufactured. This amount adds up to an inventory of annually 120 million tons globally^19^. Such high rate of accumulation has been a serious environmental problem, as RM is known to be hazardous for the environment because of its high alkalinity^20^. Obviously, recovery of RM is of great importance not only for hindering such environment effects but also for economic reasons, as it contains a large amount of metal oxides, such as Fe_2_O_3_, SiO_2_, Al_2_O_3_, and TiO_2_. For this reason, RM was considered to be utilized in various fields including ceramics^21^, construction^22^, and catalysis^23^. In catalysis, it has been mostly used as supports. For instance, RM-supported nickel^24^,^25^ and ruthenium^26^ catalysts were shown to be active for hydrogen production from ammonia decomposition. However, in general, these catalysts either did not provide high hydrogen production rates or contained expensive noble metals to reach high production rates. Actually, RM contains high amount of iron, an active metal for ammonia decomposition^13^. Thus, it can be utilized directly as catalyst for this reaction; this is the objective of this study. Here, the structure of RM was modified by a simple acid treatment to achieve a significantly high surface area with improved iron content and reduced alkali amount. After reduction at 700 °C, the resulting sample provided exceptionally high and stable hydrogen production rate for ammonia decomposition. Detailed characterization of the used catalyst unveiled that all metallic Fe species formed after the reduction step were converted into ε-Fe_2_N readily available on the surface during the reaction, suggesting that these newly formed iron nitride sites are responsible for this high and stable performance. Results presented here offer opportunities to utilize one of the key hazardous industrial wastes as an environmentally friendly, efficient, and almost cost-free catalyst for CO_x_-free hydrogen production from ammonia decomposition.

## Results and Discussions

**Figure 1:**
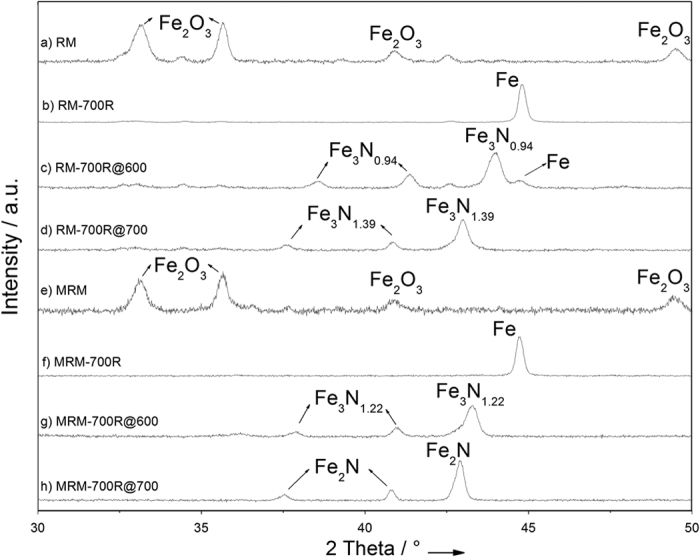
XRD pattern of (**a**) RM, (**b**) RM-700R, (**c**) RM-700R@600, (**d**) RM-700R@700, (**e**) MRM, (**f**) MRM-700R, (**g**) MRM-700R@600, and (**h**) MRM-700R@700 between 30–50°. Data for the complete range is provided in SI.

**Figure 2:**
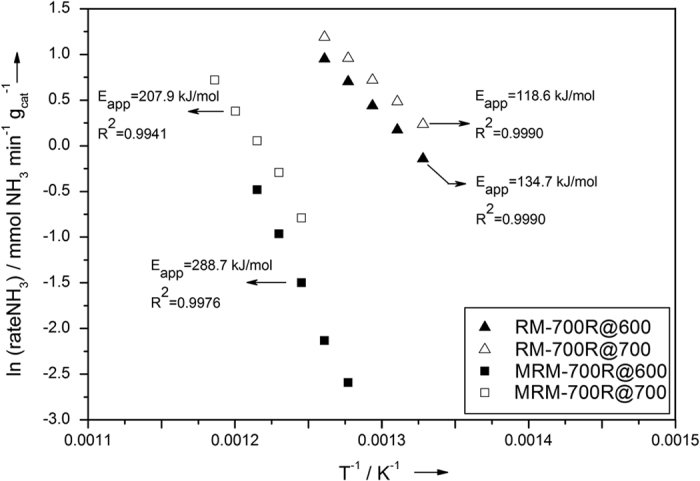
Arrhenius plots for RM-700R@600, RM-700R@700, MRM-700R@600, and MRM-700R@700 measured under differential conversions.

**Figure 3:**
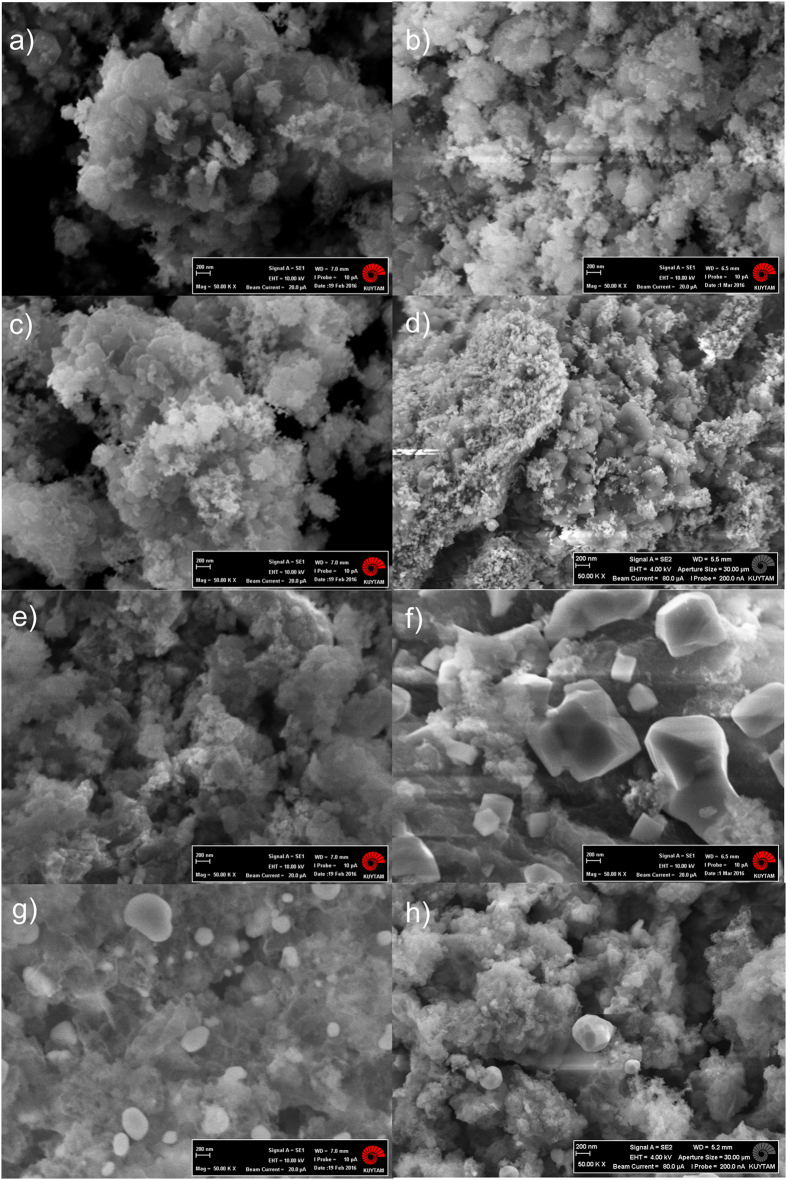
SEM images of (**a**) RM, (**b**) RM-700R, (**c**) RM-700R@600, (**d**) RM-700R@700, (**e**) MRM, (**f** ) MRM-700R, (**g**) MRM-700R@600, and (**h**) MRM-700R@700. The KUYTAM logo in images is published with permission from KUYTAM.

Temperature programmed reduction (TPR) of as-received RM (Eti Seydişehir Aluminum Factory, Konya-Turkey, contents are listed in Table S1 in Supplementary Information, SI) indicates that the conversion of iron oxide into bulk Fe is completed upon reduction in hydrogen at temperatures exceeding 650 °C (Figure S1 in SI) consistent with the results of Costa *et al*.^27^ and with the TPR of Fe_2_O_3_ as shown in Figure S2, SI. X-Ray diffraction (XRD) of RM reduced at 700 °C for 2 h in flowing hydrogen (RM-700R) confirms the conversion of iron oxides into metallic iron as indicated by the intense peak at 44.7° (Fig. 1b). RM-700R was then tested for ammonia decomposition reaction in a once-through ½″-quartz tube reactor in pure ammonia flow at atmospheric pressure and 500 °C. Data illustrated that RM-700R converts ammonia into hydrogen and nitrogen at a conversion of 5.1% for a space velocity of 42 000 NH_3_ cm^3^ h^−1^ g_cat_^−1^ at 500 °C after activation in NH_3_ flow for 1 h at 600 °C (this activated RM-700R sample is denoted as RM-700R@600). Figure 2 presents the Arrhenius plot obtained on RM-700R@600 in a temperature range from 480 to 550 °C measured under differential conversion conditions (at NH_3_ conversions below 10%). The apparent activation energy (E_app_) was measured as 134.7 kJ/mol. This value is consistent with the literature data reported for other iron-based catalysts^28^. XRD of the used RM-700R@600 catalyst given in Fig. 1c shows that the metallic iron was still present together with some newly-formed iron nitride species (Fe_3_N_y_) indicated by 2θ peaks at 38.7°, 41.4°, and 44.0° (XRD results for a complete 2θ range of 10–90° are provided in Figure S3, SI). These results indicate that activation at 600 °C for 1 h was not sufficient to convert all metallic iron into iron nitride. Thus, on a different run a fresh RM-700R sample was activated at 700 °C in 100 ml/min NH_3_ flow for 10 h (named as RM-700R@700). NH_3_ conversion on RM-700R@700 increased to 6.8% under the same conditions. The apparent activation energy measured for RM-700R@700 in a temperature range of 480 to 550 °C was 118.6 kJ/mol, slightly lower than that of RM-700R@600. Scanning electron microscopy (SEM) images, however, do not show any change in the morphology of RM-700R@600 or RM-700R@700 (Fig. 3c,d). On the other hand, XRD of RM-700R@700 show that iron is only present in Fe_3_N_y_ form. The absence of metallic iron indicates that its conversion into iron nitride was complete during the activation at 700 °C. It is also noted that the value of *y* in Fe_3_N_y_ nanoparticles increases from 0.94 to 1.39 (as evidenced by a decrease in the peak positions in XRD^29^) upon increasing the activation temperature from 600 to 700 °C accompanied by an increase in activation time from 1 to 10 h. This increase in *y* in Fe_3_N_y_ also indicates a higher degree of nitriding in RM-700R@700 than in the case of RM-700R@600^29^.

**Figure 4:**
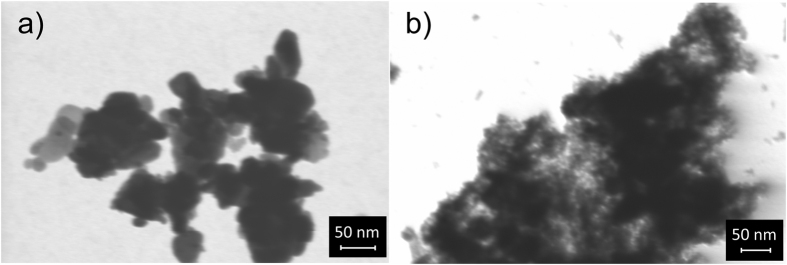
Bright field FE-SEM image of (**a**) RM and (**b**) MRM taken with an STEM detector on an FE-SEM.

In a recent study, we illustrated that the morphology and chemical composition of RM can be tuned by simple acid treatments^30^. For instance, upon 6M HCl digestion at 220 °C (modified RM, MRM), surface area of RM increased from 18 to 232 m^2^/g, while Fe_2_O_3_ content was increasing from 37.2 to 39.8 wt%. These results were further confirmed by N_2_-sorption measurements indicating an at least an order of magnitude higher N_2_-sorption capacity on MRM as compared to RM at all pressures (Figure S4 and Table S2, SI). For instance, N_2_ adsorption capacity of MRM becomes approximately 18 times higher than that of RM at a pressure of 35 bar. SEM images illustrate that MRM still shows the characteristic morphology of RM with much smaller granules present (Fig. 3e) and several rigid looking pieces are dispersed in the bulk of MRM not contained in RM (Figure S5a, SI). These are believed to be the cracked pieces of complex minerals and aluminum oxide particles in RM^31^ resulting in a higher surface area. Additional images for comparison of the morphology of RM and MRM are provided in Figure S6, SI. On the other hand, bright field FE-SEM images taken with an STEM detector show that the MRM has a highly porous structure, whereas RM presents large bulky particles (Fig. 4 and additional images in Figure S7, SI). Other details on chemical composition and textural properties of MRM in comparison with RM are given in Supplementary Tables, Tables S1 and S2, in SI.

XRD (Fig. 1f) and energy-dispersive X-ray spectroscopy (EDX) results of MRM reduced at 700 °C (MRM-700R, Figure S13, SI) confirm that iron oxide species were converted into metallic iron upon reduction. Although, SEM images indicate that RM-700R appears as similar to RM, MRM-700R shows substantial differences in morphology than MRM with mostly cubical structures present (Fig. 3b,f). These structures are well-dispersed with different sizes on the surface of MRM-700R. This substantial difference in the morphologies of the reduced samples suggests a difference in catalytic performance as iron species seem more accessible in the MRM-700R than in the case of RM-700R. Catalytic activity testing of MRM-700R at 500 °C after activating in NH_3_ flow at 600 °C for 1 h (MRM-700R@600) indicates that hydrogen production rate on this catalyst was lower than that on RM-700R. The Arrhenius plot (Fig. 2) obtained under differential conversions indicates an E_app_ of 288.7 kJ/mol. XRD of MRM-700R@600 given in Fig. 1g indicates the conversion of metallic iron into Fe_3_N_y_ species (peaks at 37.9, 41.0, and 43.3° indicate a *y* value of 1.22). After activated in NH_3_ flow at 700 °C for 10 h (MRM-700R@700), the corresponding E_app_ was measured as 207.9 kJ/mol. This decrease in E_app_ was accompanied by a shift in the XRD peak positions of Fe_3_N_y_ species to 37.5, 40.8, and 42.9°. These peaks at lower 2θ angles correspond to a *y* value of 1.5 in Fe_3_N_y_ (ε-Fe_2_N) (Fig. 1h). Formation of saturated ε-Fe_2_N species indicates that the nitriding process was more complete when the MRM-700R was activated at a higher temperature for a longer time period. The exceptionally high E_app_ values measured on these catalysts suggest that the performance of MRM-700R samples are extremely sensitive to temperature.

**Figure 5:**
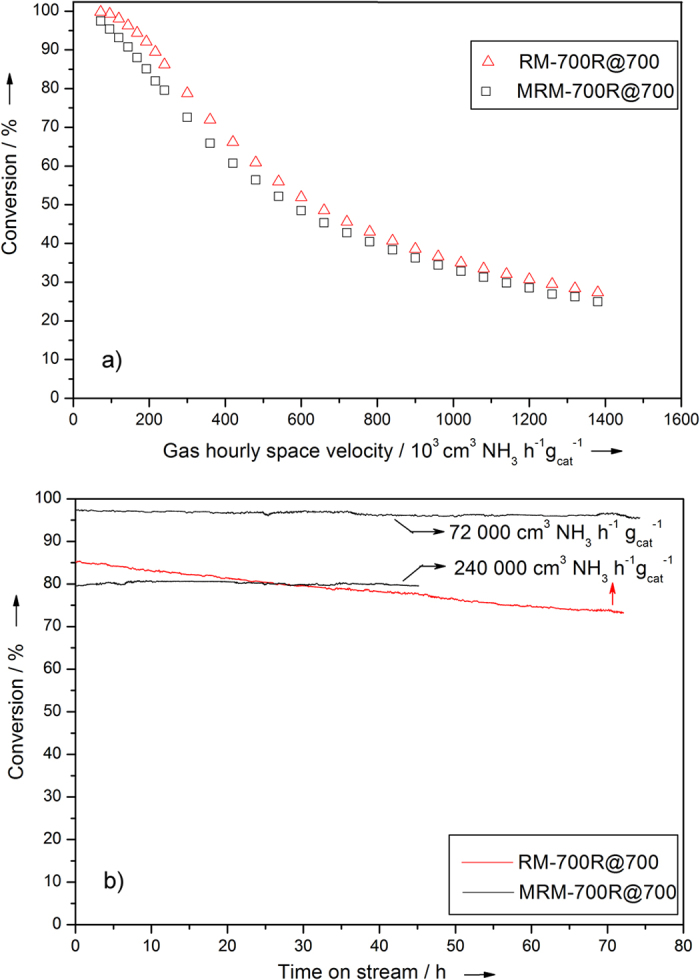
(**a**) Catalytic activity of RM-700R@700 and MRM-700R@700 at 700 °C at different space velocities, (**b**) Catalyst stability measurements at high conversions at indicated space velocities for RM-700R@700 (in red) and MRM-700R@700 (in black). Data exclude the induction period of approximately 10 hours (it is given in SI).

As discussed before, non-noble metal catalysts require high temperatures due to the slow recombinative desorption of adsorbed nitrogen atoms, which cannot take place at temperatures lower than 550 °C^32^. Thus, RM-700R and MRM-700R were activated and tested for ammonia decomposition at 700 °C (as RM-700R@700 and MRM-700R@700, respectively). Data given in Fig. 5 illustrate that ammonia conversion decreased gradually on RM-700R@700 from an initial value of 86.2 to 73.3% within 72 h at a significantly high space velocity of 240 000 cm^3^ NH_3_ h^−1^ g_cat_^−1^. However, under the same conditions, MRM-700R@700 did not show any deactivation and the conversion was remained constant at 80 ± 0.5% for more than 45 h. Thus, it is inferred that MRM-700R@700 provides a superior performance over RM-700R@700 in terms of stability, although RM-700R@700 can convert slightly higher amount of NH_3_ for short time periods as illustrated in Fig. 5. The corresponding change in H_2_ production with space velocity is provided in Figure S16, in SI. To the best of our knowledge, these results presented for MRM-700R@700 in Fig. 5 and Figure S16 are the highest ever reported for a non-noble metal catalyst for stable ammonia decomposition. For instance, Comotti *et al*.^16^ reported an iron phthalocyanine-based catalyst providing a constant conversion at approximately 75% at a space velocity of 60 000 cm^3^ NH_3_ h^−1^ g_cat_^−1^. On MRM-700R@700, a similar conversion can be achieved at almost 5-times of that space velocity, 284 000 cm^3^ NH_3_ h^−1^ g_cat_^−1^. The performance of MRM-700R@700 is even higher than an iron-based core-shell catalysts^33^ reported to be stable at 750 °C with a space velocity of 120 000 cm^3^ NH_3_ h^−1^ g_cat_^−1^ maintaining a stable ammonia conversion of 80%. As illustrated in Fig. 5, MRM-700R@700 provides the same ammonia conversion at a temperature 50 °C lower and at a space velocity twice higher than the corresponding reported values. Furthermore, MRM-700R@700 outperforms Fe_2_O_3_/CMK-5 known as an iron-based catalyst with the record high performance as well. While Fe_2_O_3_/CMK-5^13^ was deactivating quickly from an initial conversion of 90% to less than 60% within approximately 16 h at a space velocity of 120 000 cm^3^ NH_3_ h^−1^ g_cat_^−1^, MRM-700R@700 maintains 93 ± 0.5% conversion at the same space velocity. The performance of MRM-700R@700 remains stable for more than 72 h at even a higher conversion of 97 ± 0.5% for a space velocity of 72 000 cm^3^ NH_3_ h^−1^ g_cat_^−1^ (Fig. 5) at the same temperature. Moreover, even though it is not a proper comparison, the performance of MRM-700R@700 was also compared with the best performing Ru catalysts^6^ by extrapolating its temperature dependent performance data to 700 °C. This estimation illustrates that MRM-700R@700 can even provide on par performance with Ru-based catalysts at this high temperature (Figure S17, SI). Although, we note that Ru-based catalyst provides advantages of low temperature operation. Further comparison with literature is provided in Table S3, SI.

Elucidation of the reasons for such high performance is challenging due to the complex nature of RM and MRM. However, as discussed above, XRD of MRM-700R@700 given in Fig. 1h show that there is no metallic iron left after the reaction; instead all iron was present in ε-Fe_2_N form. Consistent with these results, SEM image given in Fig. 3h shows that round-shaped species with varying diameters were newly formed on the surface after reaction. The EDX analysis (Figure S15, SI) performed on these round-shaped species confirms the XRD results and indicates that these round-shaped species are nitrided iron (detailed images are provided in Figure S18, SI). Consistent with this observation Rohith Vinod *et al*. illustrated that nanosized iron clusters can be converted into iron nitride upon ammonia treatment at high temperatures. They also presented similar round-shaped structures by SEM imaging^34^. These results also confirm why the apparent activation energy on MRM-700R was higher than that on RM-700R. Because, as illustrated before^35^, totally nitrided iron particles (ε-Fe_2_N) require a higher activation energy than a mixture of iron with partially nitrided iron does. Thus, it is inferred that the reduced iron species observed as well-dispersed cubical structures in Fig. 3f were converted into ε-Fe_2_N during the induction period of 10 h in NH_3_ flow at 700 °C (Figure S19, SI). The evidence for this conclusion was the presence of isolated round-shaped particles in the SEM of MRM-700R after reaction (MRM-700R@700 in Fig. 3h), also confirmed by EDX and XRD results. It is noted that such ball-like iron nitride species were not observed on the SEM images of RM-700R@700 (Fig. 3d) even though its XRD results indicate the presence of Fe_3_N_1.39_ in the absence of any metallic iron. These results suggest that iron nitride species formed under the activation conditions are trapped inside the bulk of RM in RM-700R@700. Apparently, a significant increase in surface area caused by the acid digestion makes iron species readily available. Upon activation at 700 °C, they can be converted into ε-Fe_2_N in MRM-700R@700. ε-Fe_2_N has hexagonal close-packed (hcp) structure with much larger lattice constants than other iron nitrides. Thus, trapped iron species in RM-700R@700 can not be converted into ε-Fe_2_N because of space limitations, instead they were partially saturated to form Fe_3_N_1.39_. Based on these differences between RM-700R@700 and MRM-700R@700, it is concluded that the readily available ε-Fe_2_N species in MRM-700R@700 are directly responsible for stable hydrogen production at such a high rate. It is also noted that metallic iron content of MRM reaches to 35.4 wt% after reduction at 700 °C. Such high and stable catalytic performance can also be due to the synergistic effect of this high Fe content with the presence of alkali elements, such as Na and K, in trace amounts remained after acid digestion (Table S1, SI). These alkali elements are known for promoting the performance of a non-noble metal catalyst^36^. Their presence in higher amounts in RM-700R@700 might be the reason for higher initial activity on RM-700R@700 as compared to MRM-700R@700. Sintering of these and/or other components present in RM might be blocking the pores under the reaction conditions, making the trapped iron nitride species unavailable for the reaction. This blocking of the pores can be the reason for deactivation observed on RM-700R@700. Whereas MRM-700R@700 does not suffer from this problem as saturated ε-Fe_2_N species are readily available on the surface as demonstrated by the SEM images.

## Conclusions

In summary, we show that RM can be simply converted into an exceptionally active and stable catalyst for hydrogen production from ammonia. Results illustrate that MRM converts ammonia into hydrogen and nitrogen at constant conversion for more than 72 h at 700 °C. For instance, stable hydrogen production rates were measured as 72 and 196 mmol H_2_ min^−1^ g_cat_^−1^ for the corresponding space velocities of 72 000 and 240 000 cm^3^ NH_3_ h^−1^ g_cat_^−1^, respectively, at that temperature. These values are significantly higher than the data reported on any other non-noble metal catalysts and on par with the estimated performance of Ru-based catalysts at 700 °C. Characterization of the used MRM catalyst indicated the presence of ε-Fe_2_N moieties readily available on the surfaces as the active species for the reaction. Considering that we are utilizing a hazardous industrial waste, being produced in vast amounts, these results provide opportunities towards an eco-friendly, efficient, stable, and almost cost-free catalyst for CO_x_-free hydrogen production from ammonia.

## Methods

### Materials and sample preparation

MRM was prepared by acid digestion of RM in 6M HCl at 220 °C, as described elsewhere^30^. In summary, RM was treated with HCl 37% (Merck) in a microwave digester (Milestone Microwave Digestion System (SK-10) with temperature control, operating at 350W) and precipitated by aqueous ammonia solution (25%, Merck). 25 g of red mud was mixed with 100 ml distilled water followed by addition of 150 ml 6M HCl solution. The solution was digested in a digester for 45 minutes at 220 °C. While stirring the resulting acid treated solution, aqueous ammonia was added until a pH of 8 was reached. The precipitated RM was recovered by centrifugation. After washing the sample several times with distilled water, the sample was dried overnight at 110 °C.

### Materials characterization.

#### X-Ray diffraction (XRD)

For XRD measurements a Bruker D8 Discover X-Ray Diffraction system with a Cu Kα_1_ radiation source employing a wavelength of 1.5418 Å was used. A Vantec-1 detector was used with a slit of 1 mm and the power rating of X-ray generator was set to 40 kV and 40 mA. The 2θ range of the measurements were between 10–90° with a step size of 0.01263°. A double sided tape was used to fix approximately 10 mg of sample on a glass slide. This glass slide was placed on a gum to attach it to a sample holder. Background signal generated by the tape was subtracted during investigation of the phases. ICDD PDF-4 2014 database was used for phase identification.

#### Brunauer-Emmett-Teller (BET) pore volume and surface area analysis

For the BET analysis a Micrometrics ASAP 2020–Physisorption Analyzer was used. For each measurement, approximately 400 mg sample was used. The sample was degassed under vacuum at 120 °C for 8 h before the measurement. The free space measurement was performed in He gas. The volumetric N_2_ adsorption/desorption isotherm was measured between P/P_0_ = 0.01–0.95 at cryogenic temperature. BET surface area was calculated between P/P_0_ = 0.05−0.3 using liquid nitrogen (40 data points).

##### Scanning electron microscopy coupled with energy-dispersive X-ray spectroscopy (SEM/EDX)

SEM images were obtained by a Zeiss Evo LS 15 scanning electron microscope by analyzing the samples under ultra-high vacuum with an accelerating voltage of 10 kV and working distances of 6.0–8.5 mm. A secondary electron detector was used. Images were obtained at various magnifications. EDX experiments were performed by using Rontec xflash 1106 EDX detector. For detailed analysis some of the samples were analyzed by a Zeiss Ultra Plus field emission scanning electron microscope at magnifications higher than 50 000×. Under ultra-vacuum, an accelerating voltage of 5 kV and working distance of 3.1 mm, a secondary electron detector was used for obtaining these images. An XFlash 5010 EDX detector with 123 eV resolution was used for EDX imaging on this microscope. Bright field images were taken in transmission mode of the FE-SEM using a high resolution STEM detector.

##### X-Ray fluorescence (XRF) spectroscopy

For the determination of elemental composition a Bruker S8 Tiger XRF spectrometer in standardless mode under helium atmosphere with 18 mm mask was used. Powder samples were loaded into a XRF sample cup (Chemplex Industries Inc., Cat. No: 1430) with a thin-film support (Prolene film with 4 μm thickness, Chemplex Industries, Inc., Cat. No: 426). Typical impurities of this film are notified as Ca, P, Fe, Zn, Cu, Zr, Ti, Al in the ppm level. The loose powder method was selected for the measurements together with “Best analysis” and “Oxides” methods. SpectraPlus Eval2 V2.2.454 was used for data interpretation.

##### Temperature programmed reduction (TPR)

TPR analysis was performed by an MKS Cirrus 2 mass spectrometer connected to a Micrometrics AutoChem II 2920 instrument. The sample was exposed to 10% H_2_-90% Argon mixture and was heated up to 700 °C (with a temperature ramp of 5 °C/min) and held at that temperature for 2 h. The effluent gas stream was analyzed by passing through a heated silica capillary (heated to 120 °C) to the mass spectrometer where it was ionized at 70 eV. The mass spectra was recorded between 1–200 atomic mass units (amu) continuously, with each scan taking 13s from 1 to 200 amu. The atomic mass unit of 2 (*m/z* = 2) was analyzed for hydrogen.

#### High-Pressure Volumetric Adsorption Analysis

Adsorption measurements were carried out by a Micrometrics High Pressure Volumetric Analyzer (HPVA II). Approximately 0.4 g sample was used for measurements. For the outgassing process, samples were heated up to 100 °C and kept at 10^−6^ bar overnight. After N_2_ adsorption measurements, samples were outgassed and dry masses were weighed for both RM and MRM. N_2_ adsorption isotherms were obtained by considering the dry masses of the samples.

### Catalytic Performance Measurements

Reduction and catalytic activity measurements were performed at atmospheric pressure in a once-through ½″-quartz tube reactor placed in a Carbolite split-tube furnace. The sample was placed between two quartz wool layers and fixed in the reactor. The catalysts were reduced at 700 °C in H_2_ (99.9999%, Linde) for 2 h. After the reduction step, the catalyst was purged with He (99.999%, Messer) for 45 min at 700 °C before starting the pure ammonia (99.99%, Linde) flow. Ammonia conversion was measured online by a Hiden QGA gas analyzer. Calibration for H_2_, N_2_, and NH_3_ gases and online measurements were performed at an ionization potential of 70 eV and at a sampling pressure of 3.4 × 10^−6^ Torr. Ammonia decomposition rate per gram of catalyst per time was calculated by multiplying the space velocity with the ammonia conversion. Ammonia conversion was kept below 10% to maintain the differential conversion conditions for the activation energy measurements so that a constant NH_3_ concentration can be sustained throughout the catalyst bed. For these measurements approximately 0.15 g sample was used and the temperature was varied in a temperature range of 480–550 °C following the activation in NH_3_ flow at specified condition. For the activity measurements at high ammonia conversion conditions, 25 mg of the catalyst sample was mixed with fumed silica at a ratio of 1:40, and this mixture was used. The blank experiments were performed to confirm that the reactor, fumed silica, and quartz wool layers do not provide any ammonia conversion at the conditions considered. Measurements performed at different space velocities and catalyst particle sizes show no effect of mass transfer limitations and illustrate that the data produced are perfectly reproducible.

## Additional Information

**How to cite this article**: Kurtoğlu, S. F. and Uzun, A. Red Mud as an Efficient, Stable, and Cost-Free Catalyst for CO_x_-Free Hydrogen Production from Ammonia. *Sci. Rep.* **6**, 32279; doi: 10.1038/srep32279 (2016).

## Supplementary Material

Supplementary Information
