# Supplementary material for: Red Mud as an Efficient, Stable, and Cost-Free Catalyst for COx-Free Hydrogen Production from Ammonia
Source: Sci Rep. 2016 Aug 25;6:32279. doi: 10.1038/srep32279 (PMC4997713; doi:10.1038/srep32279)
Supplement: Supplementary Information [file srep32279-s1.pdf]

Supplementary Information

Red Mud as an Efficient, Stable, and Cost-Free Catalyst for CO_x-Free Hydrogen Production from Ammonia

Samira Fatma Kurtoğlu[a,b], Alper Uzun[a,b,*]

[a]Department of Chemical and Biological Engineering, Koc University,Rumelifeneri Yolu, Sariyer, 34450, Istanbul, Turkey;

[b]Koç University TÜPRAŞ Energy Center (KUTEM), Koç University Rumelifeneri Yolu, Sariyer 34450, Istanbul, Turkey;

**Corresponding Author: auzun@ku.edu.tr*

Additional Characterization Data

TPR Curve of RM

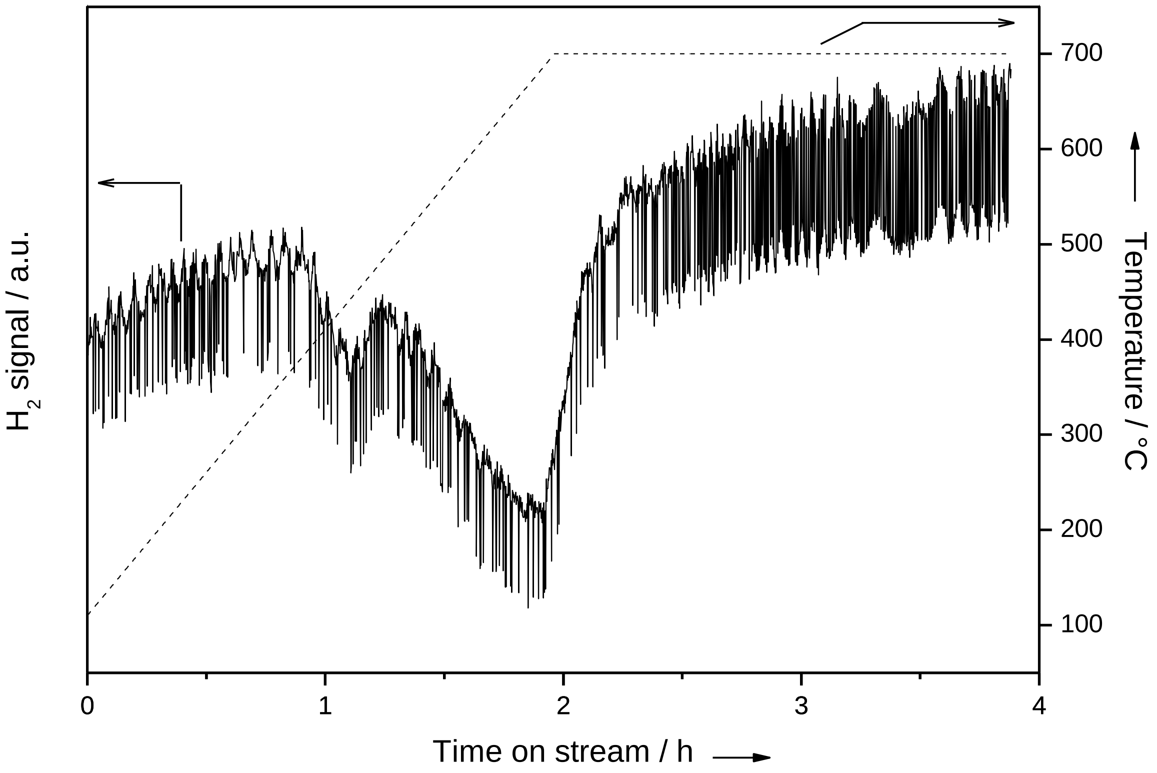

***Figure S1*. TPR curve of RM.**

According to Figure S1, there are two main reduction steps. The first one at 442 °C signifies the Fe_3O_4 formation from Fe_2O_3. The second broad peak appearing after 500 °C is related to the FeO formation from Fe_3O_4 and further reduction of FeO to Fe[1]. After 700 °C H_2 signal becomes stable and reduction is completed with the product of metallic iron.

TPR Curve of Fe_2O_3

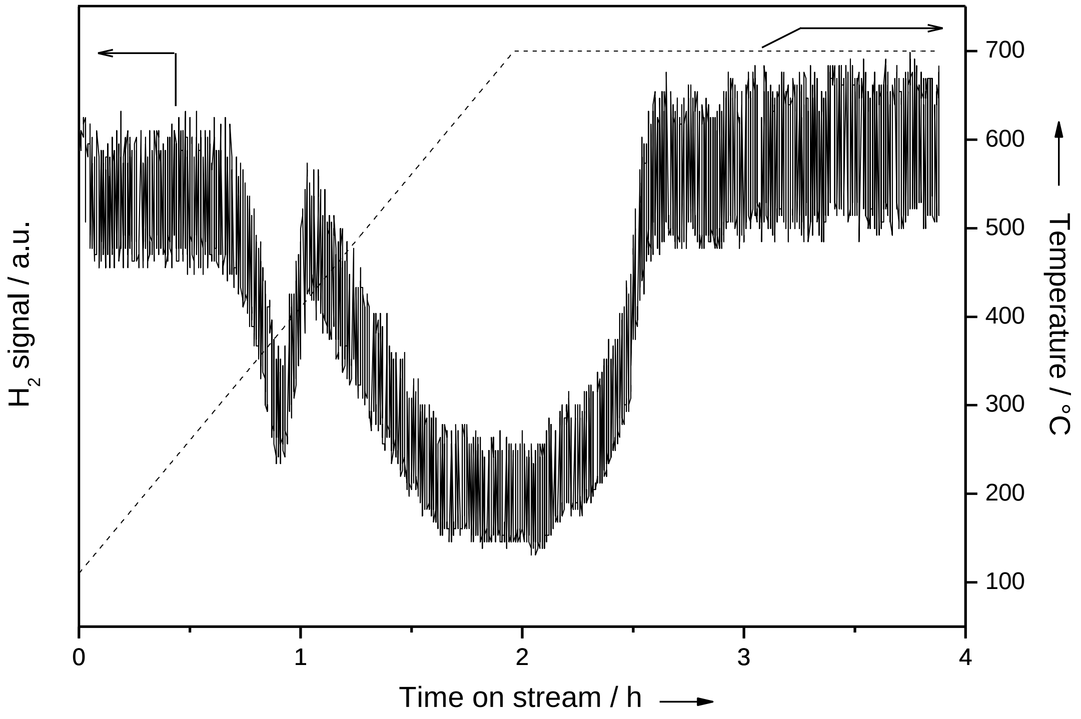

Figure S2. TPR curve of pure Fe_2O_3

According to Figure S2, similar to RM, the first peak starting at 388 °C signifies the phase change from Fe_2O_3 to Fe_3O_4[2]. The second broad peak shows the second reduction step of Fe_3O_4 to Fe, by two different steps as discussed for RM in Figure S1.

XRD Pattern for the 2Θ Range 10-90°

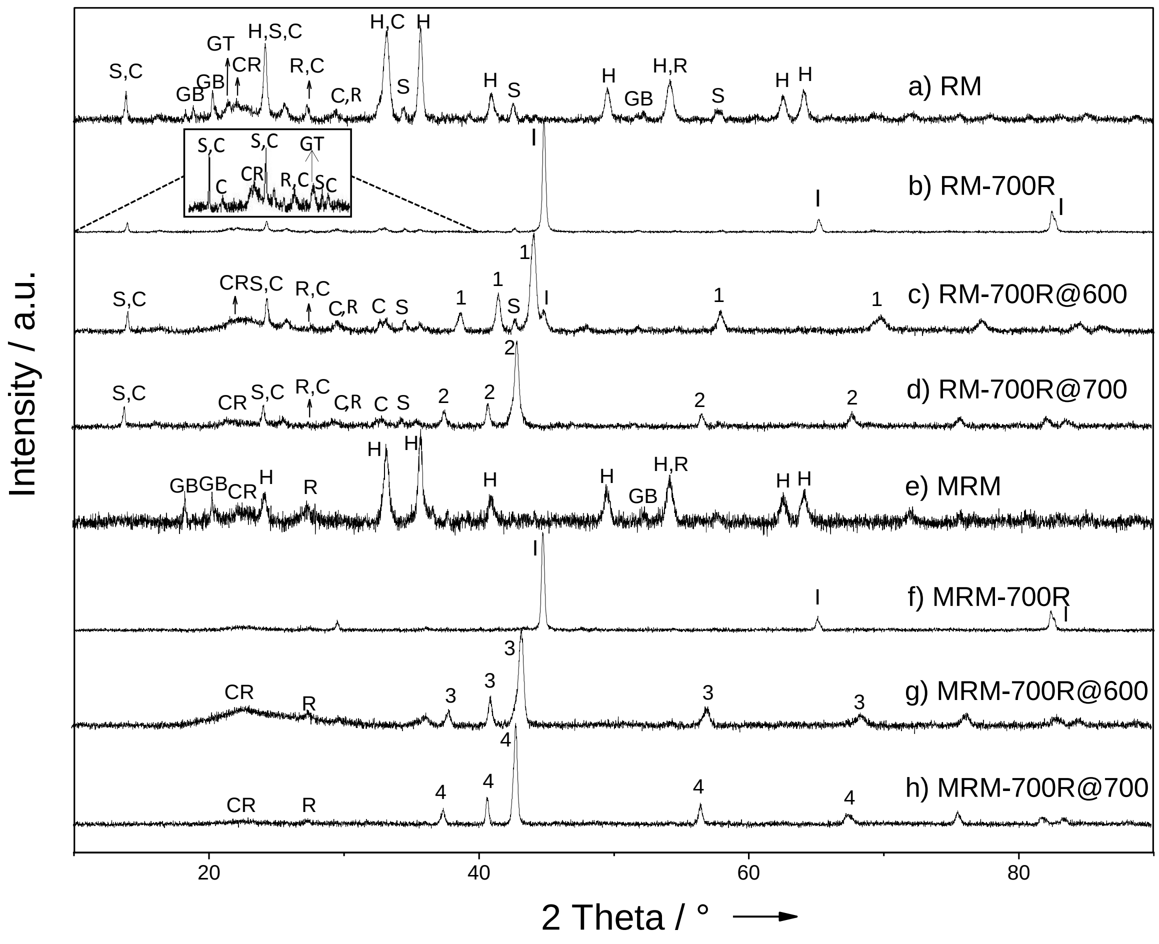

Figure S3: XRD pattern of a) RM, b) RM-700R, c) RM-700R@600, d) RM-700R@700, e)MRM, f) MRM-700R, g) MRM-700R@600 and f) MRM-700R@700 H–Hematite (Fe_2O_3), S–Sodalite ($K_{1.4}Na_{5.3}Ca_{1.2}Al_6(SiO_4)_6(CO_3)_{0.3}(SO_4)_{0.8}Cl_{0.9}$), C–Cancrinite ($Na_6(Al_6Si_6O_{24}))(CaCO_3)_2$), GB–Gibbsite ($Al(OH)_3$), GT–Goethite ($FeO(OH)$), R–Rutile ($TiO_2$), CR–Cristobalite ($SiO_2$), IN–Iron Nitride ($Fe_3N$), I–Iron (Fe), iron nitrides; 1–$Fe_3N_{0.94}$, 2–$Fe_3N_{1.39}$, 3–$Fe_3N_{1.22}$, 4–Fe_2N.

Figure S3 a shows the XRD pattern of RM. Several species also confirmed by XRF (Table S1) are introduced. In Figure S3 e the pattern of MRM does not show the characteristic peak at 13.9° only designating the complex minerals sodalite (PDF 04-011-7226) and cancrinite (PDF 01-074-7052) (labelled by S and C on the figure). After the modifications mainly the following phases left: hematite, rutile, cristobalite, and gibbsite. Due to the

decomposition of Na, K, and Ca containing minerals upon acid treatment, the peaks belonging to calcite, cancrinite, and sodalite can not be detected in XRD. The peak of gibbsite is seen clearly at 18.2 and 20.2°. It is inferred that the aluminum phases which were present in sodalite and cancrinite precipitated as aluminum hydroxides during the addition of ammonia to the acid treated slurry. Cristobalite is a phase which does not undergo any phase change during acid treatment. Moreover, the XRF results presented in Table S1 further supports this interpretation. Since RM becomes amorphous after modification it is expected that some peaks become less visible than those of hematite because of the presence of intense features of Fe_2O_3. The strong peak at 44.7° (PDF 00-006-0690) of the patterns of RM-700R and MRM-700R given in Figure S3 c and f, respectively, shows the conversion of hematite to metallic iron. After reduction of RM-700R, it is possible to detect the phases also present in RM as, sodalite, cancrinite, cristobalite, rutile, and goethite. Because complex minerals like sodalite and cancrinite are decomposed and metallic iron species provide strong features, only metallic iron peak could be detected in MRM-700R (Figure 3,f). However, as shown by XRF (Table S1) some other species (for example, rutile, cristobalite) are still left after modification.

RM-700R@600 (Figure S3 c) shows metallic iron peak at 44.7° together with peaks 38.7, 41.4, and 44.0° corresponding to $Fe_3N_{0.94}$ (PDF-01-077-9851). The iron nitride phase in RM-700R@700 (Figure S3 d) is $Fe_3N_{1.39}$ indicated by the main peaks at 37.6, 40.8, and 43.0° (PDF 01-070-7409). $Fe_3N_{1.22}$ in MRM-700R@600C (Figure S3, g) is shown by the features at 37.9, 41.0, and 43.3° (PDF 04-011-6419). A further shift of the peak of iron nitride species towards left appears in the sample MRM-700R@700 (Figure S3, h) and ε-Fe_2N formation in MRM-700R@700 is indicated by the peaks at 37.5, 40.8, and 42.9° (PDF 04-007-1467).

High Pressure Adsorption Isotherms of RM and MRM

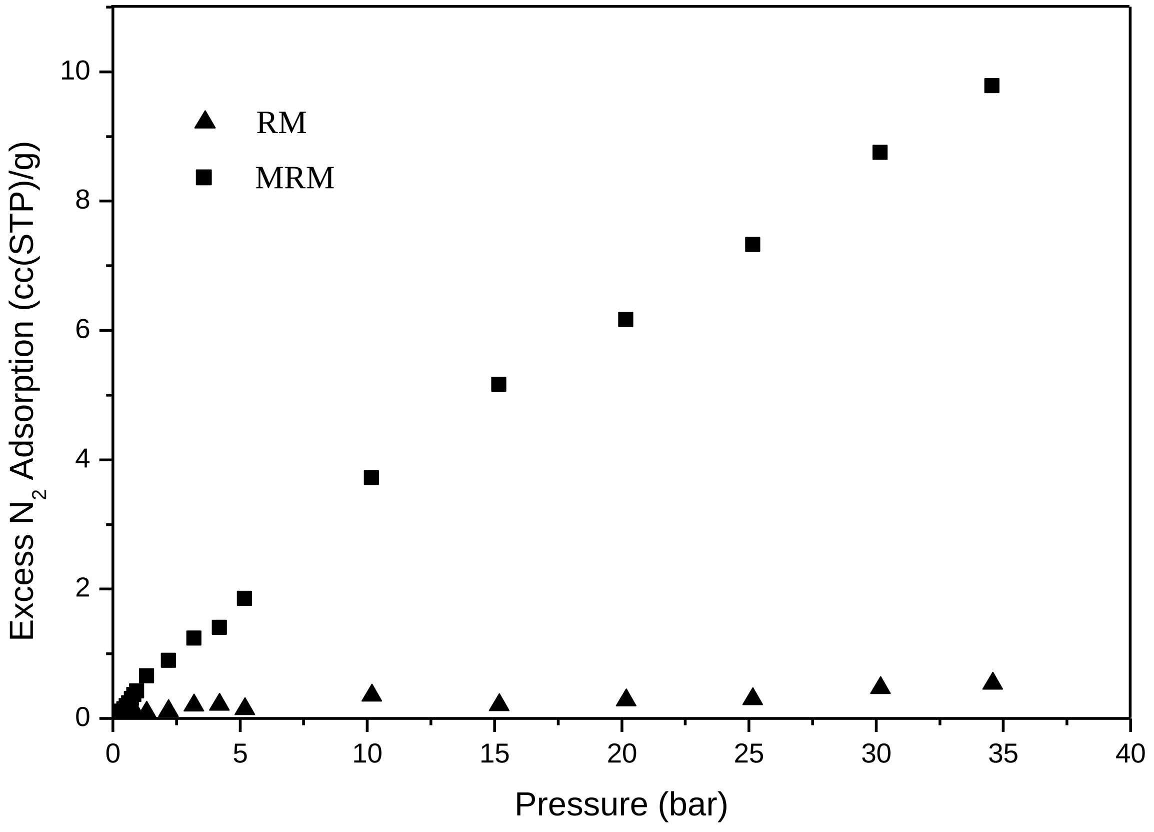

Figure S4. N_2 adsorption isotherms for RM and MRM

Figure S4 shows the N_2 sorption analysis of RM and MRM. Further numerical data can be found in Table S2.

Additional SEM images of MRM

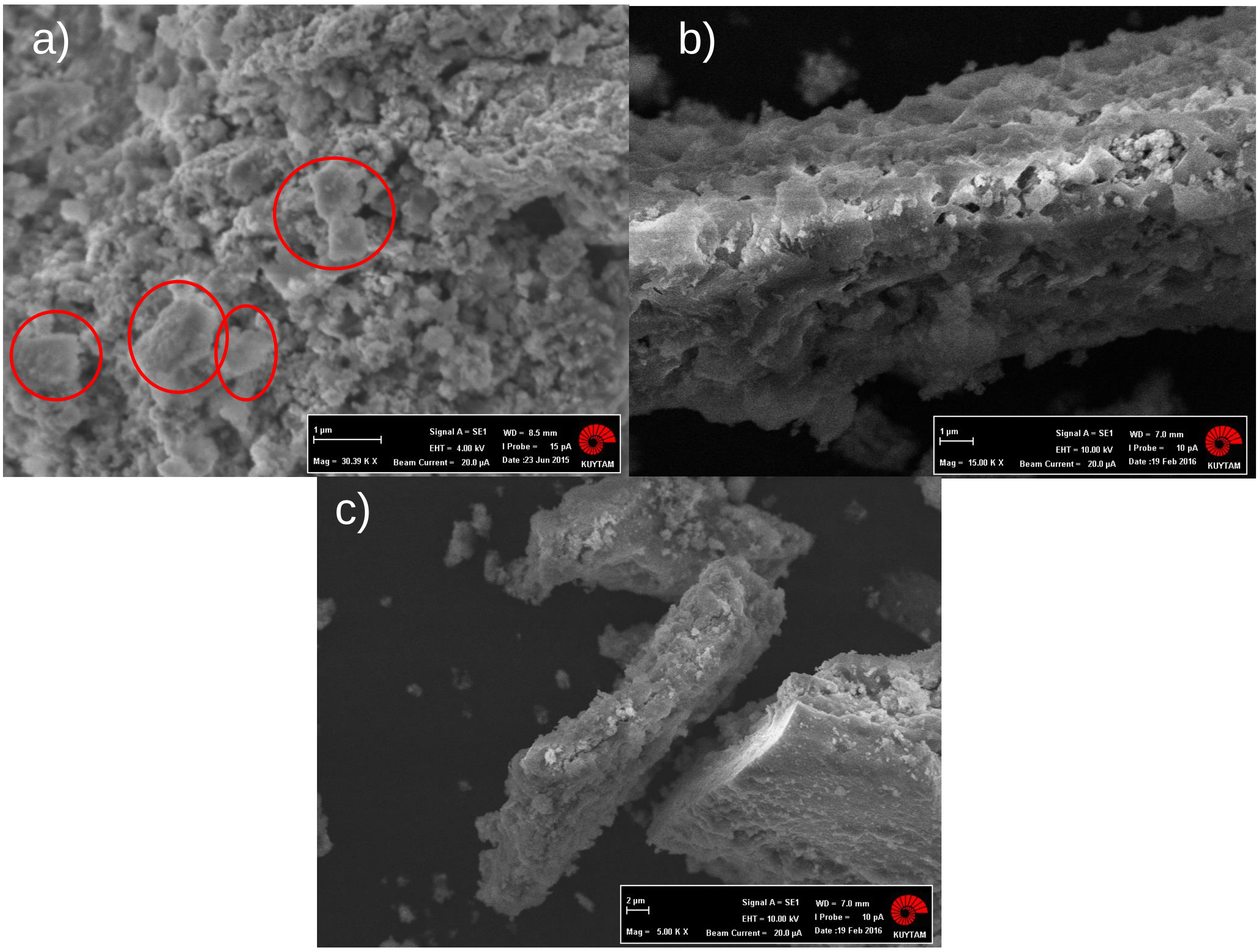

Figure S5: Additional SEM image of MRM a) some newly formed particles believed to be dissolution products of decomposed minerals, b) porous structures not present in RM, c) some particles having sharper edges not observed in RM. The KUYTAM logo in images is published with permission from KUYTAM.

Additional SEM images showing different structures upon modification is given in Figure S5.

Comparison of RM and MRM in terms of SEM Images

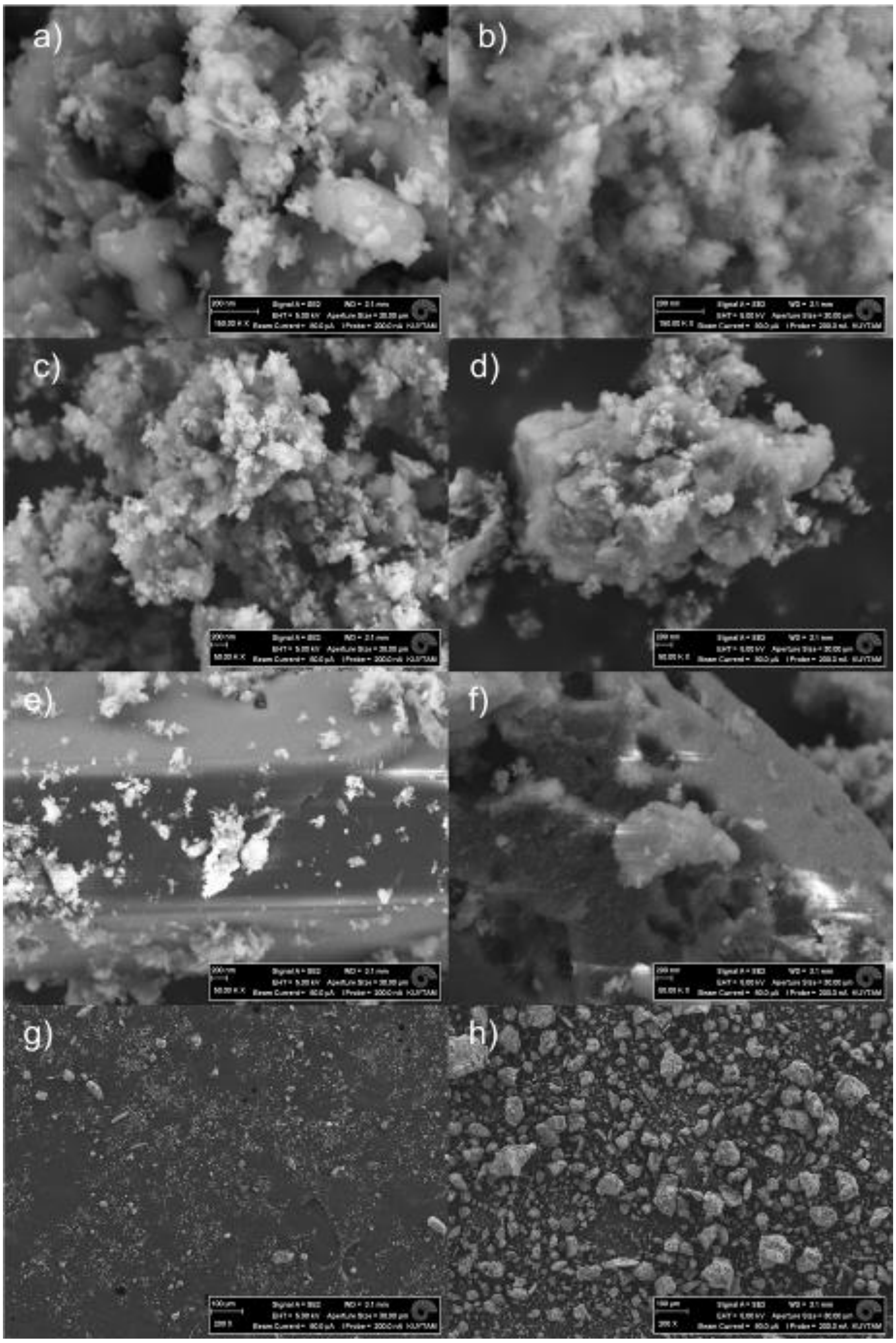

Figure S6. SEM images of a) RM at 150 000× magnification, b) MRM at 150 000× magnification, c) RM at 50 00×, d) MRM at 50 000×, e) RM at 50 000×, f) MRM at 50 000×, g) RM at 200×, and h) MRM at 200×. The KUYTAM logo in images is published with permission from KUYTAM.

As Figure S6 a and b suggest, untreated RM has a clear image at 150,000× magnification where the small particle sizes are hardly detected in MRM at the same magnification. These images show that after modification, MRM consists of smaller particles then RM which results in a higher surface area. Several additional images at various magnifications are given in Figure S6. When comparing Figure S6 e and f one can see that this specie becomes porous after modification. Also it can be noted that, the very low magnification images (Figure S6 g an h) shows a very different view between RM and MRM's bulk. MRM consists of more sharp edged rock looking particles.

Comparison of RM and MRM in terms Bright Field FE-SEM Images Taken with STEM Detector

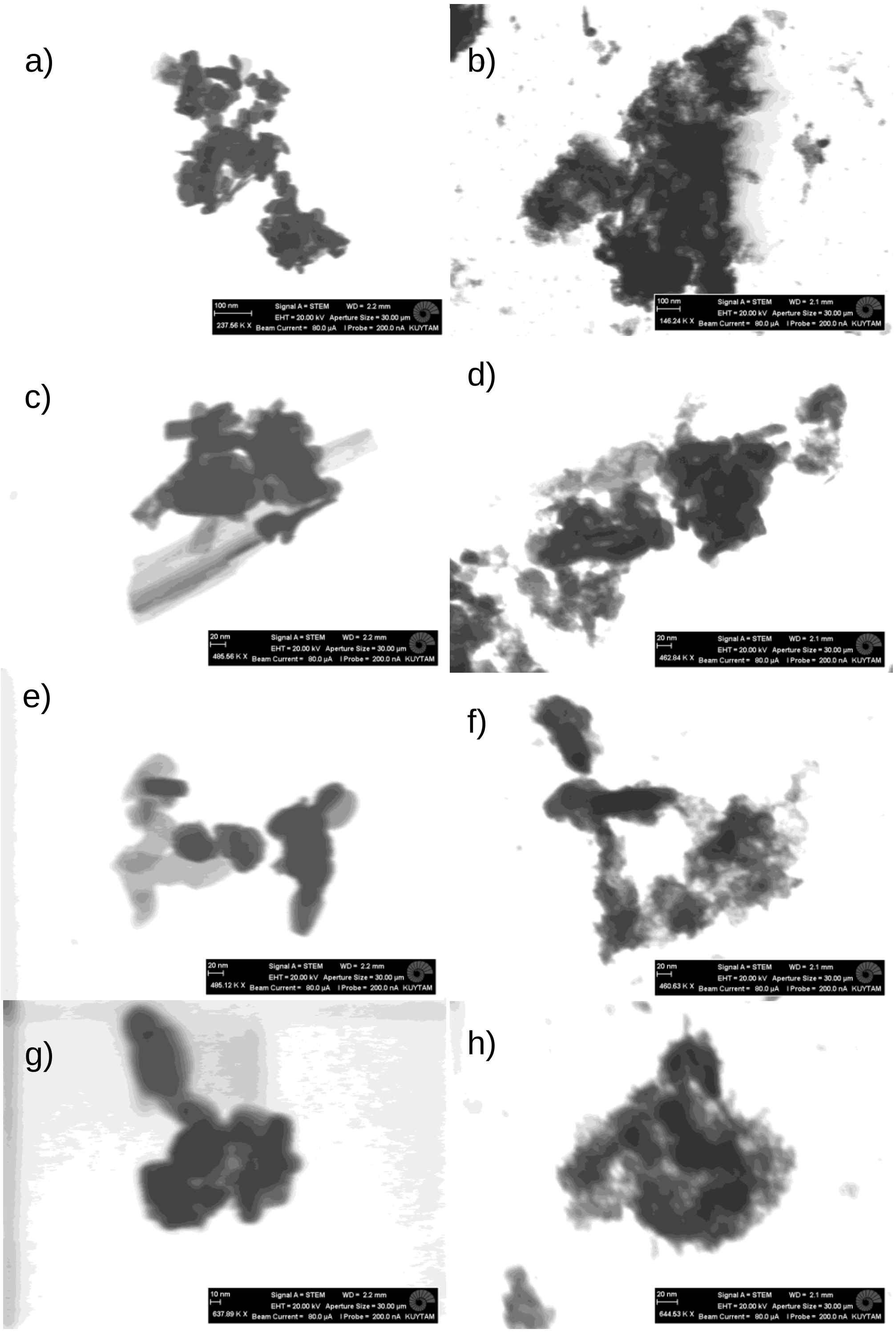

Figure S7. Bright field FE-SEM images of RM (a,c,e,g) and MRM (b,d,f,h). The KUYTAM logo in images is published with permission from KUYTAM.

Figure S7 supports the findings of SEM images in Figure S6, N_2 sorption analysis in Figure S4 and BET measurements given in Table S2 such that MRM shows a highly porous structure whereas RM appears as bulky particles.

SEM Images and corresponding EDX Spectra

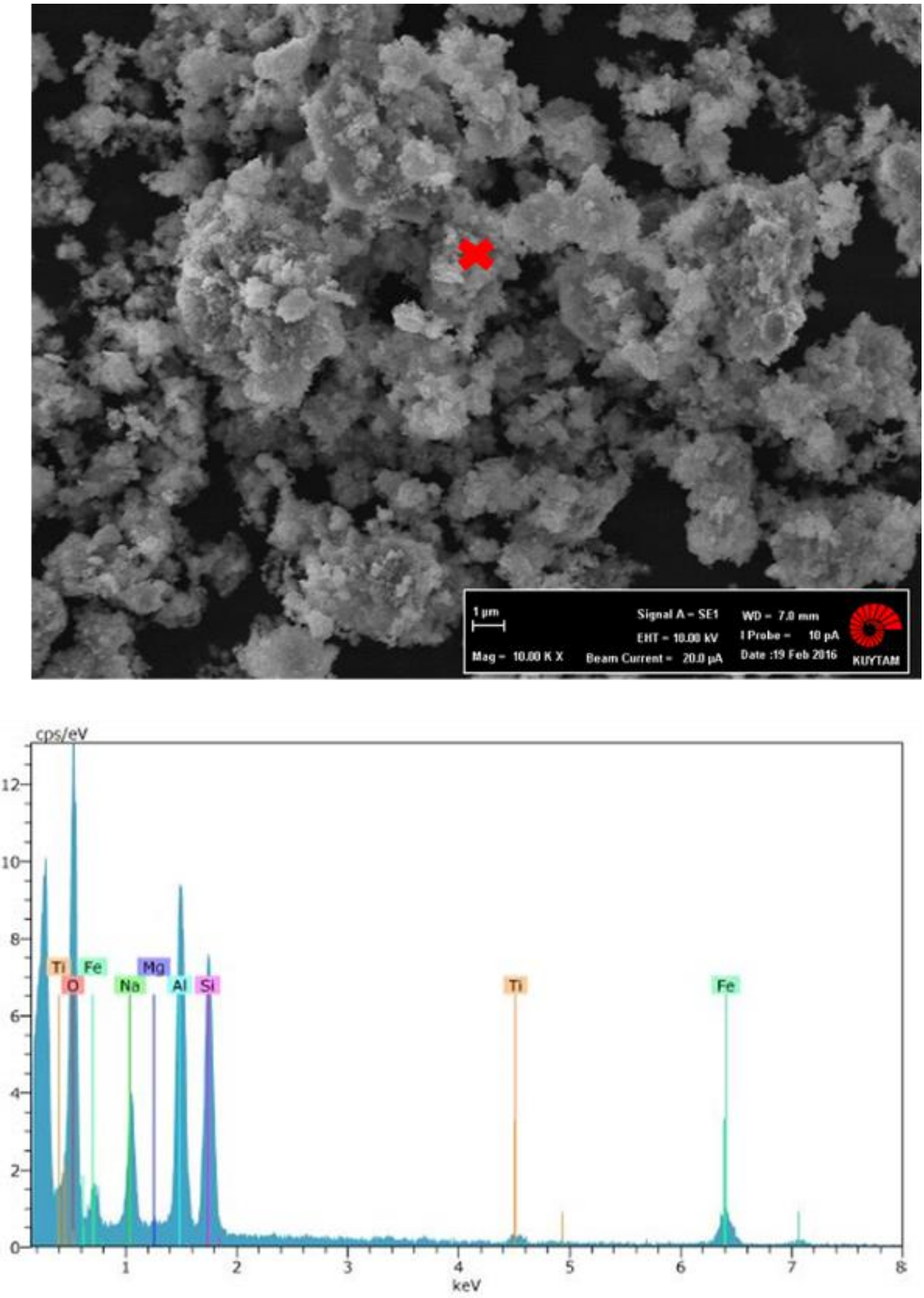

Figure S8. SEM image and corresponding EDX spectrum of RM. The KUYTAM logo in images is published with permission from KUYTAM.

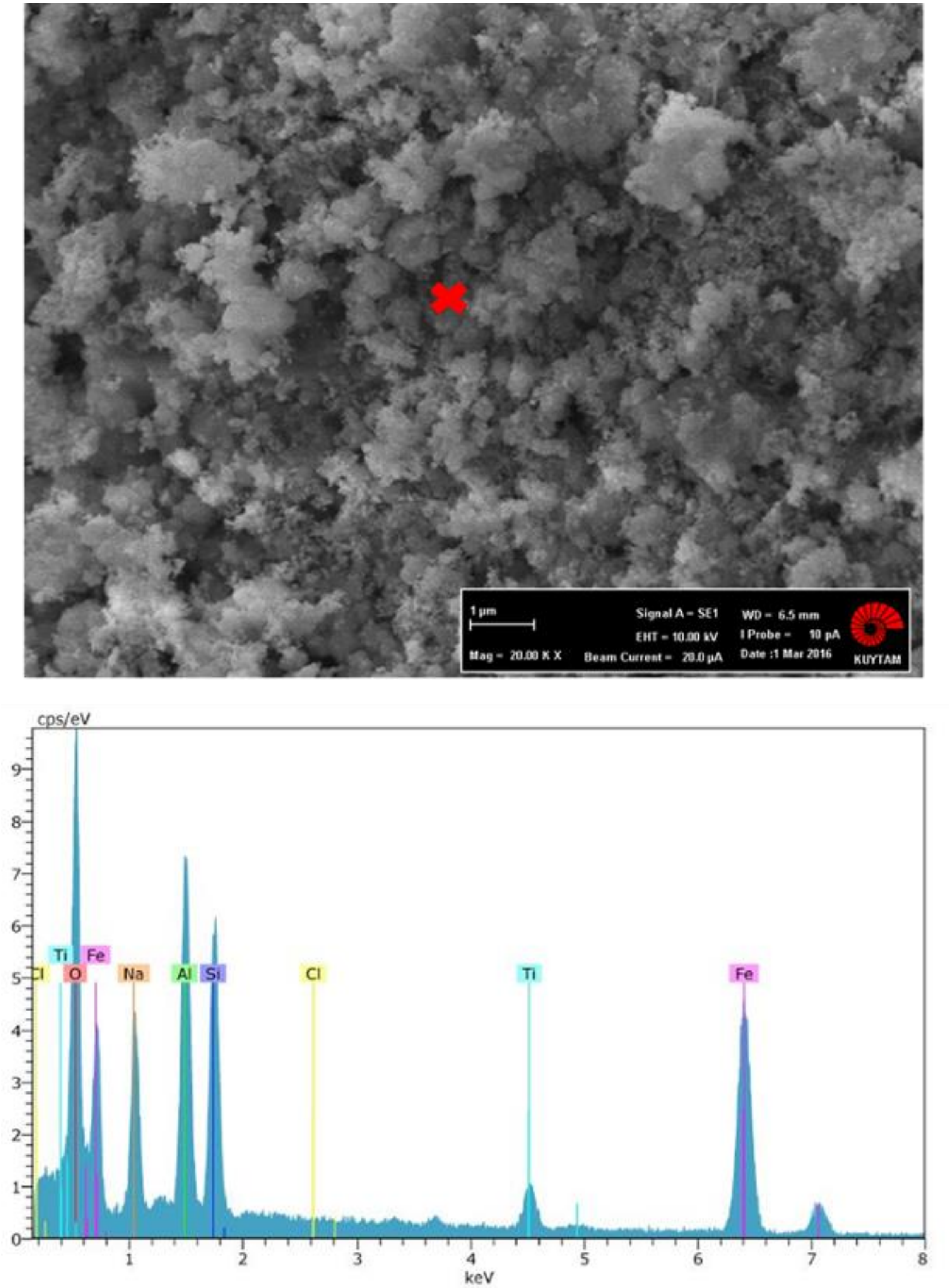

Figure S9: SEM image and corresponding EDX spectrum of RM-700R. The KUYTAM logo in images is published with permission from KUYTAM.

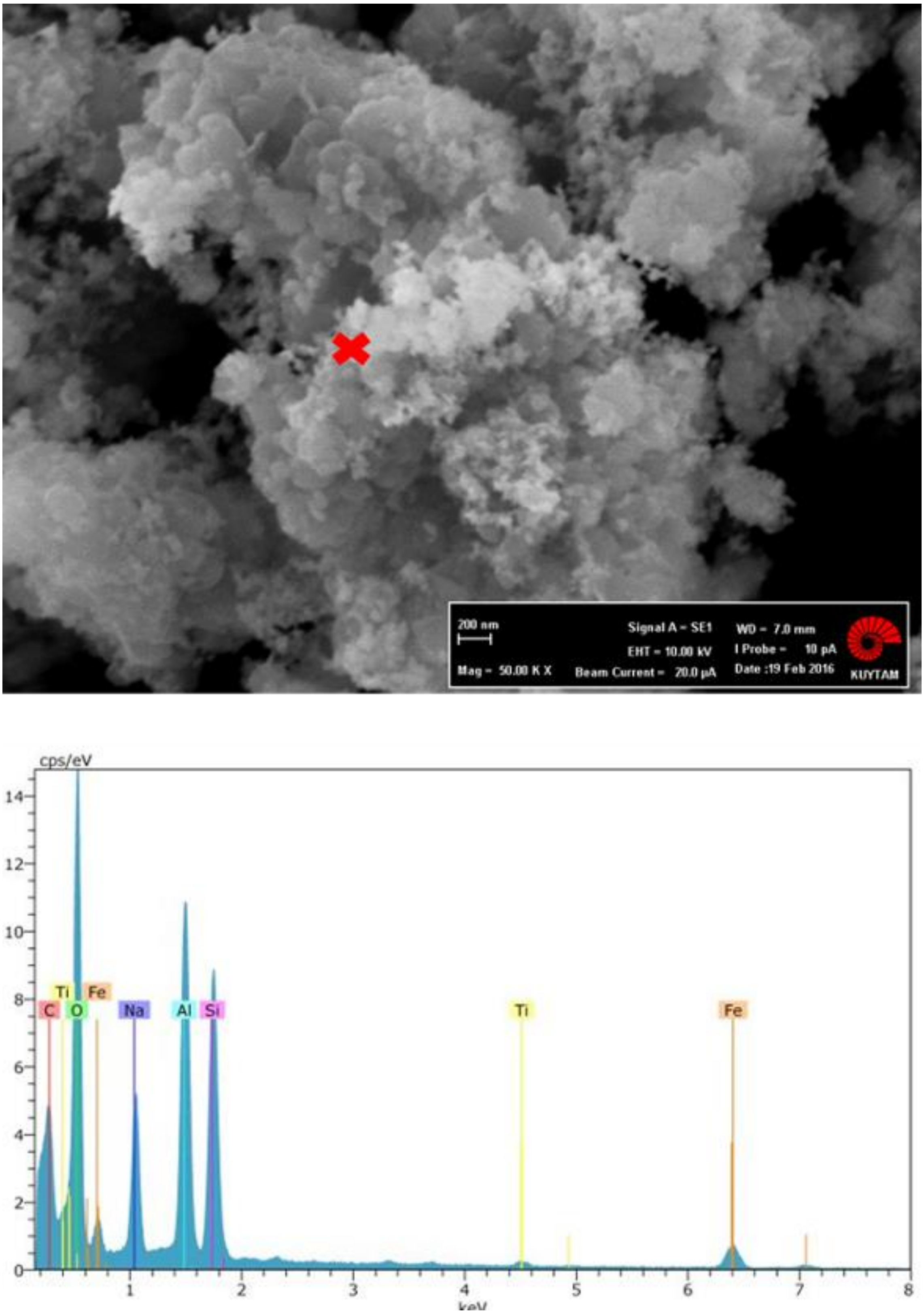

Figure S10: SEM image and corresponding EDX spectrum of RM-700R@600. The KUYTAM logo in images is published with permission from KUYTAM.

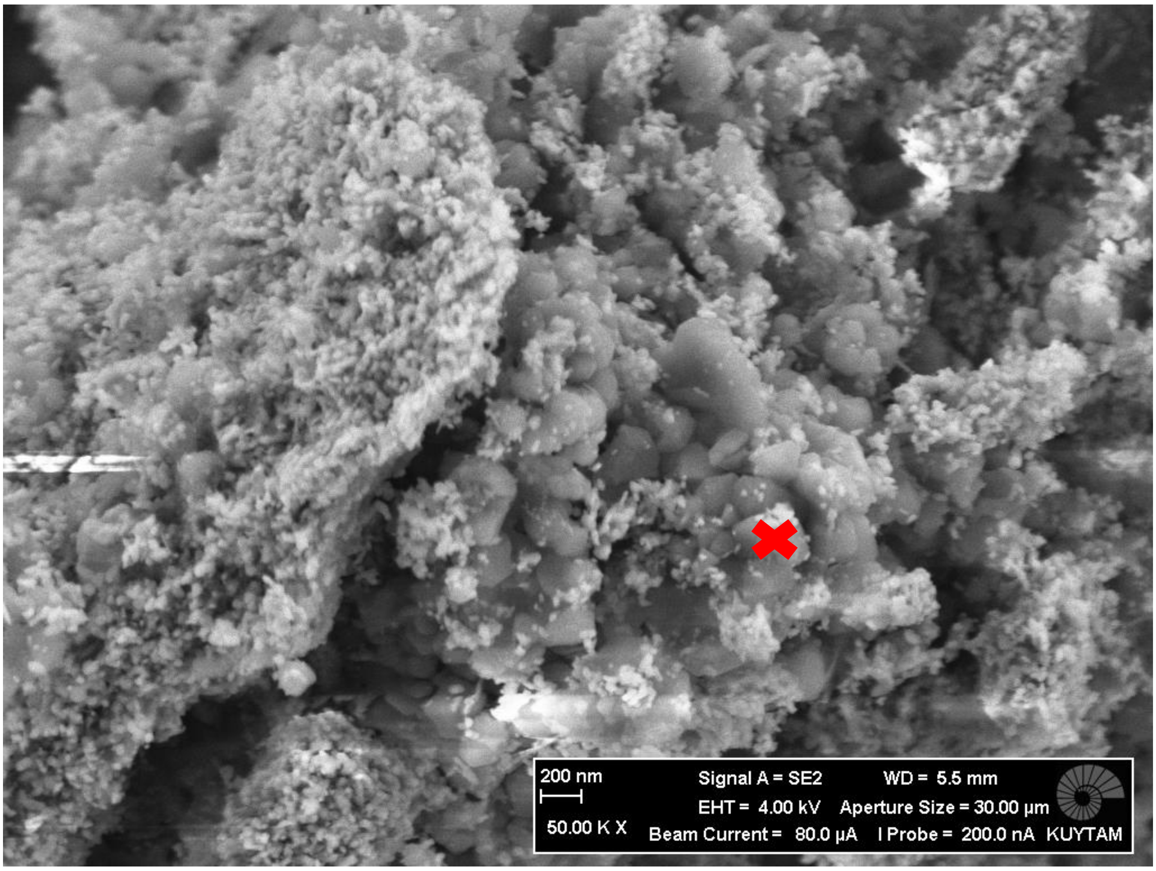

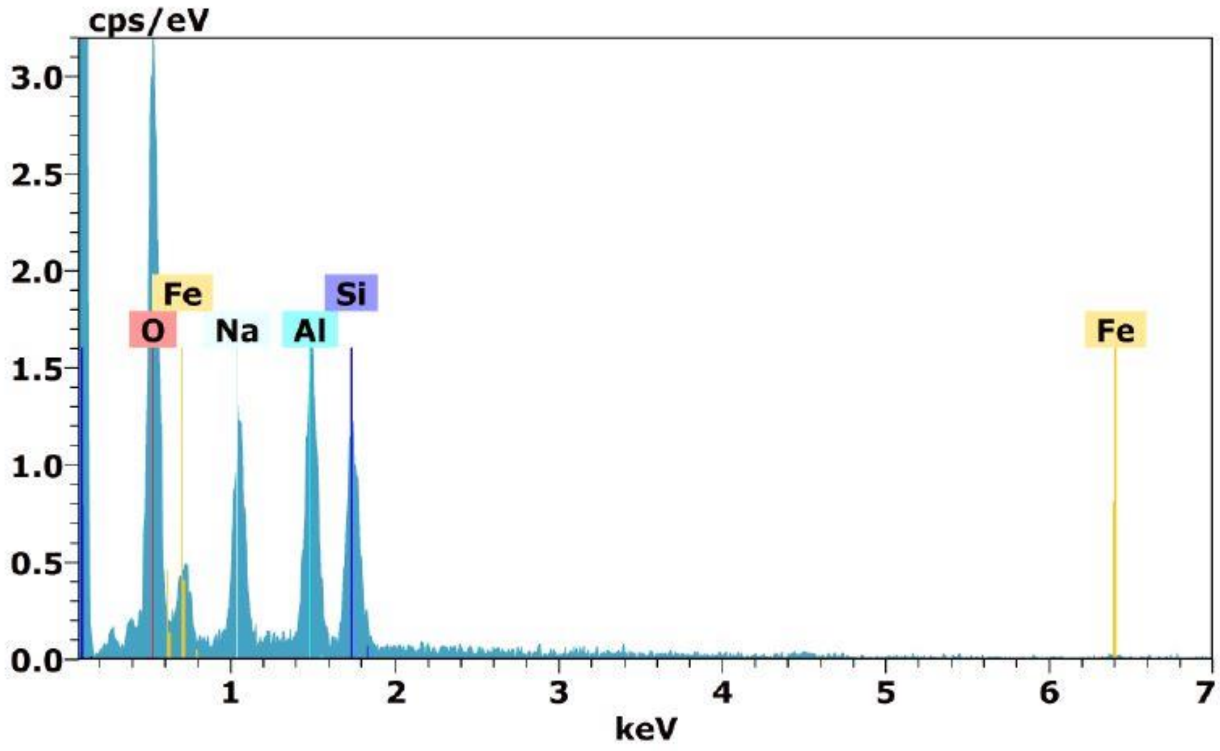

Figure S11: SEM image and corresponding EDX spectrum of RM-700R@700. The KUYTAM logo in images is published with permission from KUYTAM.

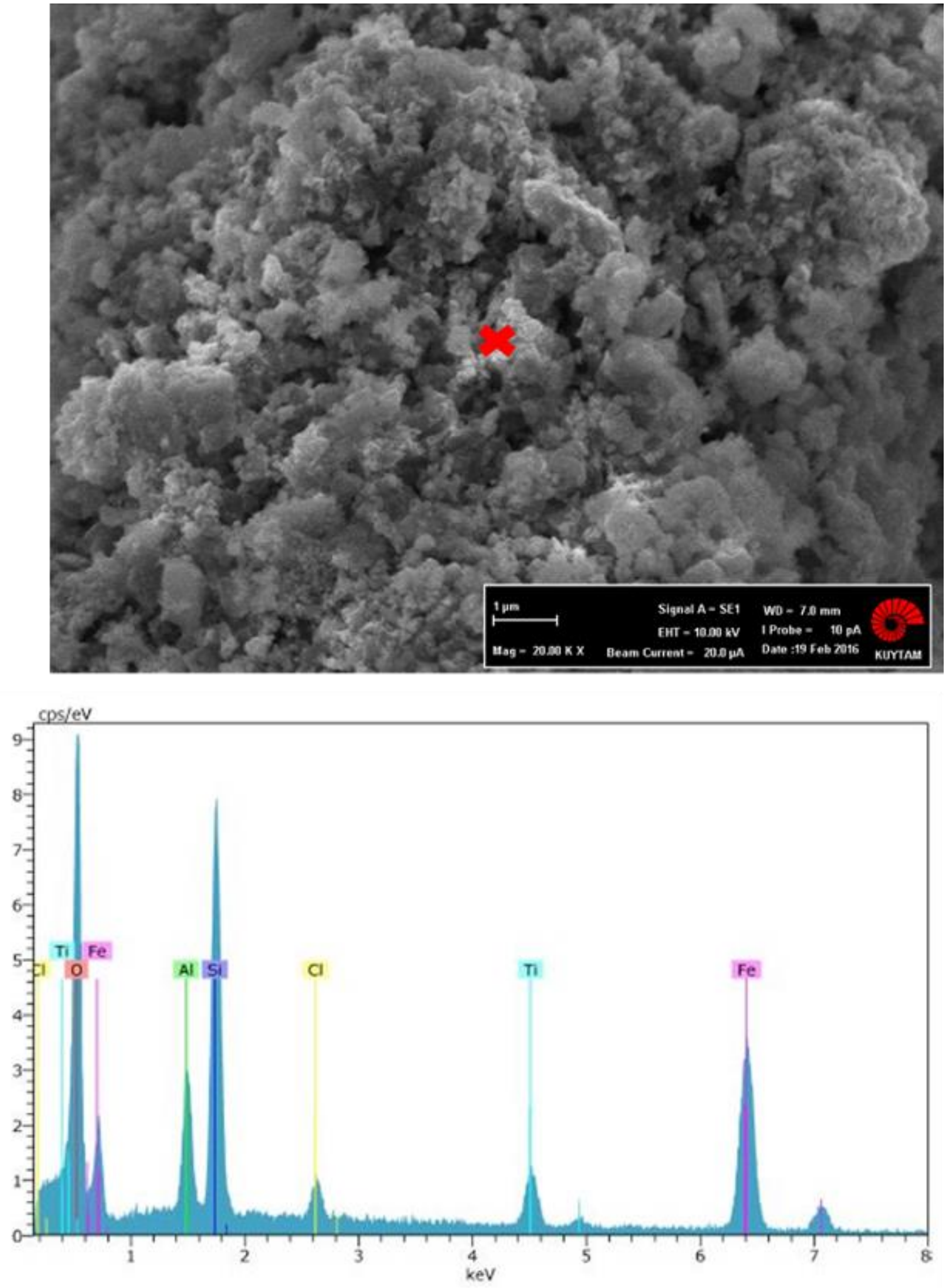

Figure S12: SEM image and corresponding EDX spectrum of MRM. The KUYTAM logo in images is published with permission from KUYTAM.

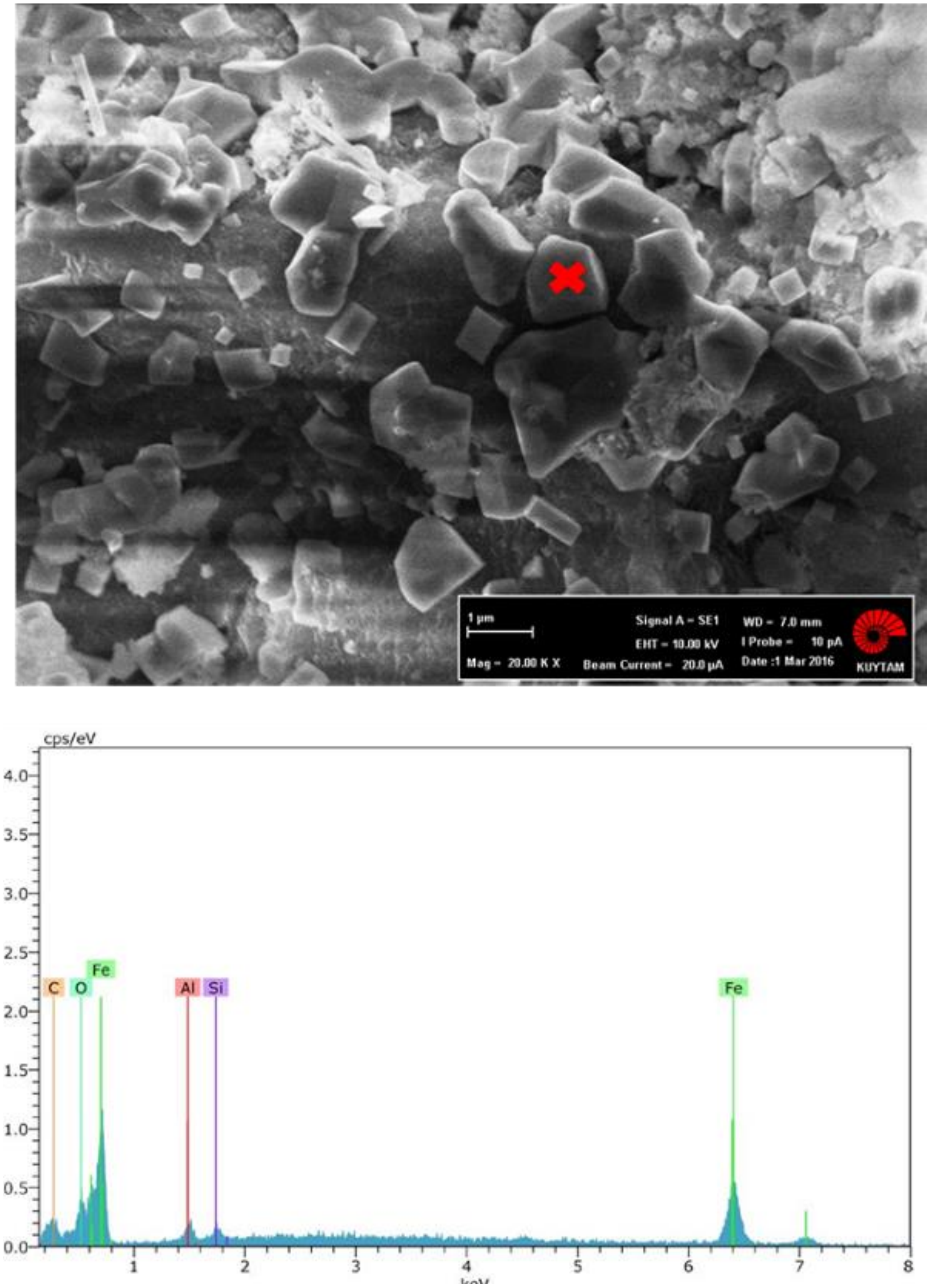

Figure S13: SEM image and corresponding EDX spectrum of MRM-700R. The KUYTAM logo in images is published with permission from KUYTAM.

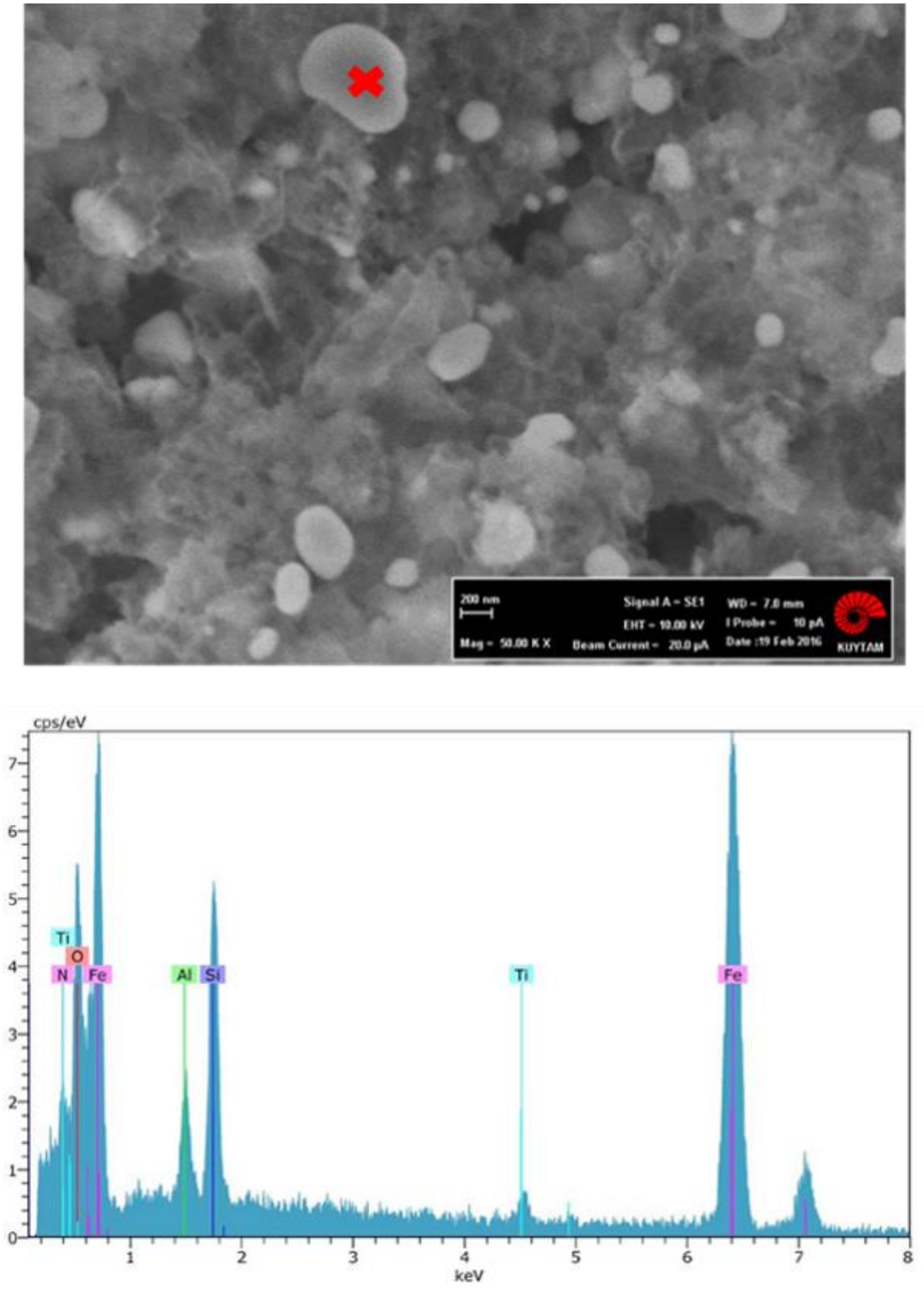

Figure S14: SEM image and corresponding EDX spectrum of MRM-700R@600. The KUYTAM logo in images is published with permission from KUYTAM.

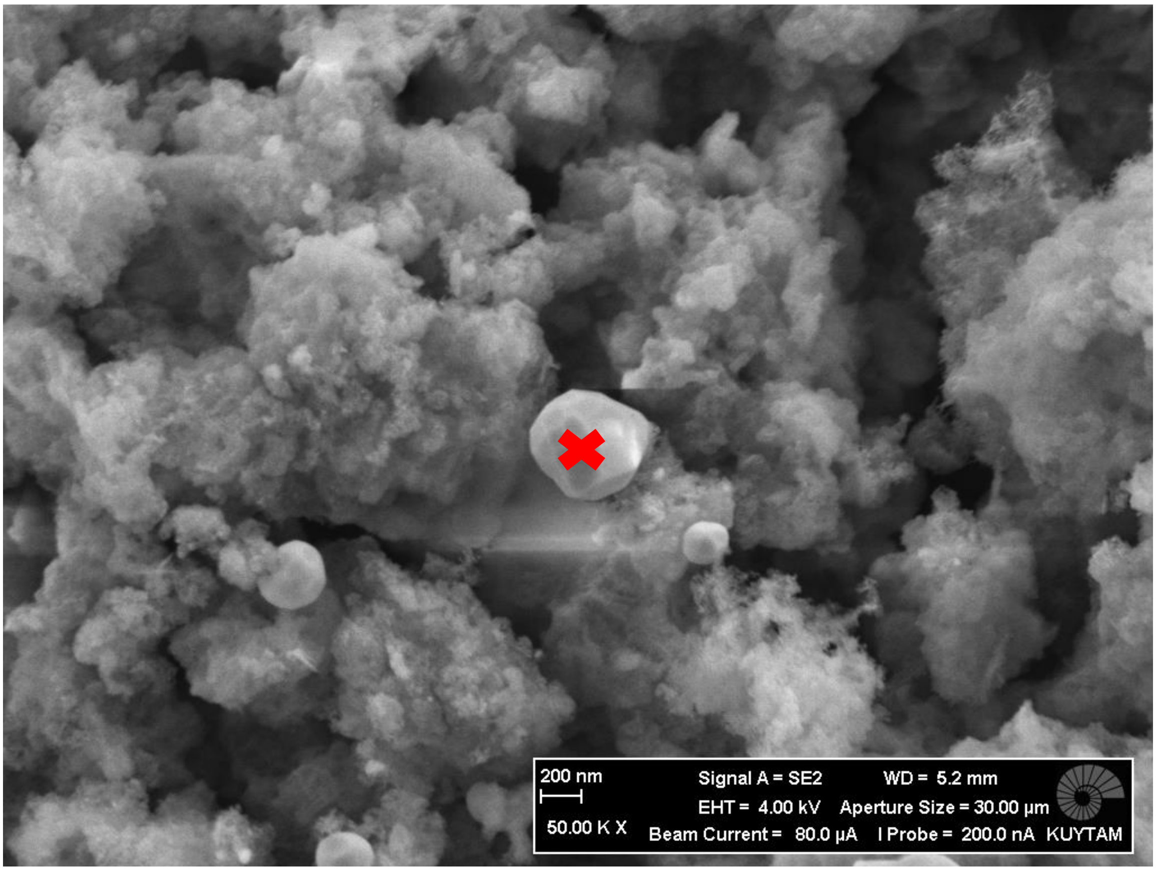

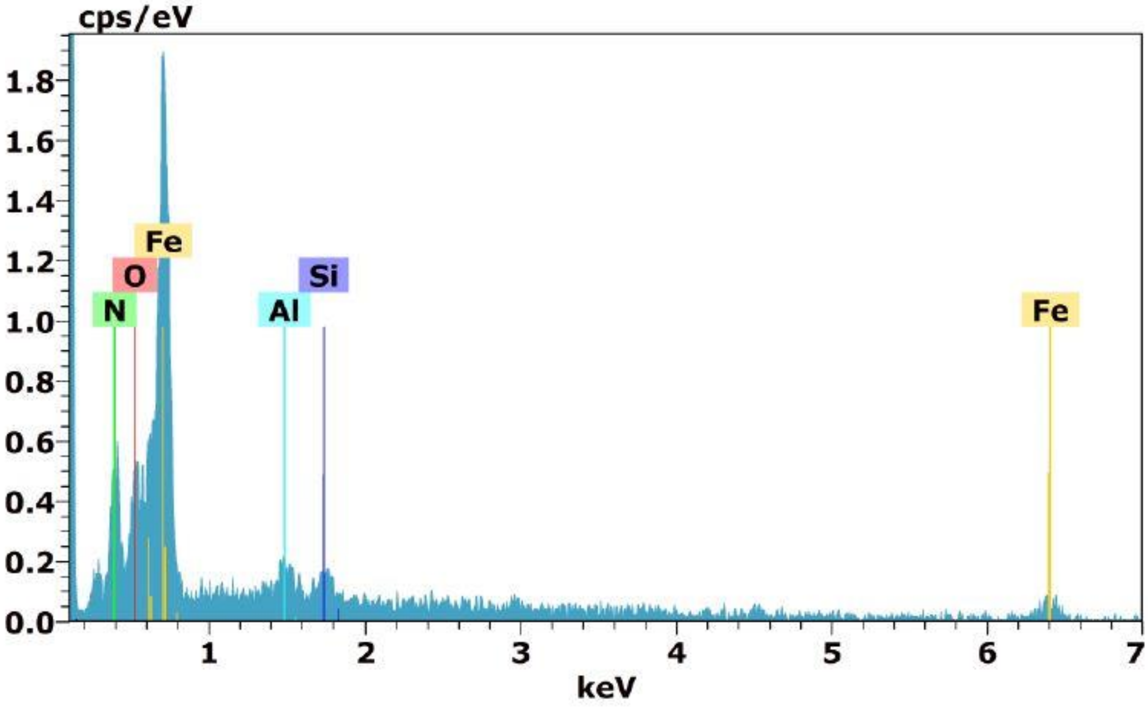

Figure S15: SEM image and corresponding EDX spectrum of MRM-700R@700. The KUYTAM logo in images is published with permission from KUYTAM.

In Figure S8, the spectrum of RM indicates nearly all components contained in RM which are also detected by XRF shown in Table S1. These main elements contained in RM are Fe, Ti, Al, Si, Na, Mg, and O, showing that the oxides of the previous elements are the main phases (also detected by XRD, Figure S3 and by XRF, Table S1). Figure S9, Figure S10, and Figure S11 are showing the EDX spectrum of RM-700R and RM-700R@600 and RM-700R@700, respectively. These three samples exhibit similar EDX spectra and similar morphology. Even though XRD indicates a complete reduction of iron species in the RM-700R, the cubical species seen in MRM-700R (Figure S9) were not observed in SEM images, most probably due to the low surface area of RM-700R. The acid treated RM (MRM) in Figure S12 shows a very similar spectrum to RM, however with a more intense peak of Cl which is increasing due to the HCl treatment. We see that in the reduced MRM sample, MRM-700R, no Cl is left when investigating the cubical structure given by a very intense peak of Fe (Figure S13). The remaining oxygen peak (very low when compared to the bulk of RM) is due to the oxides of Ti, Al, or Si remaining in MRM-700R. In Figure S14, when investigating the spherical structure we see a very intense N peak not present in any other sample proving the presence of N together with the very intense peak of Fe in MRM-700R@600. Similar to MRM-700R-600C, in Figure S15, the spherical structure is indicated as iron nitride shown by the very intense N peak in MRM-700R@700.

Activity of MRM-700R given in terms of H_2 Production

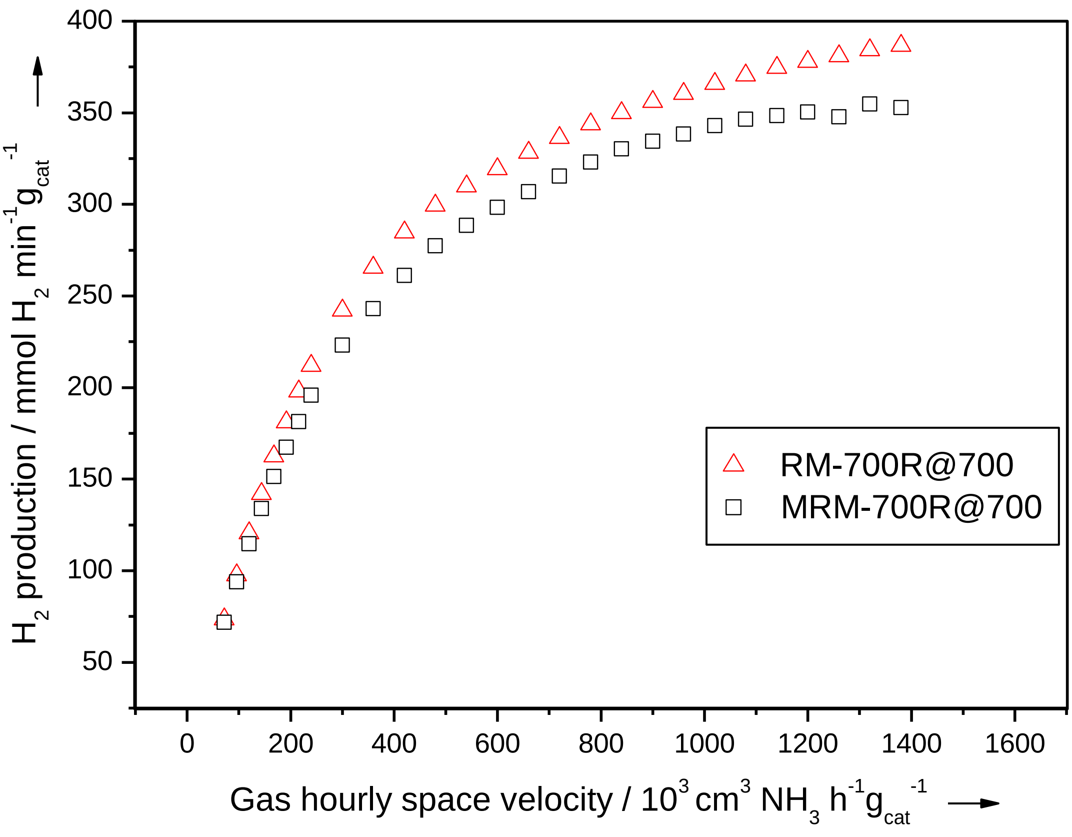

Figure S16. Variation of H_2 production rate with space velocity on RM-700R@700 and MRM-700R@700 at 700 °C.

Comparison of Ru-based Catalysts with MRM at 700°C

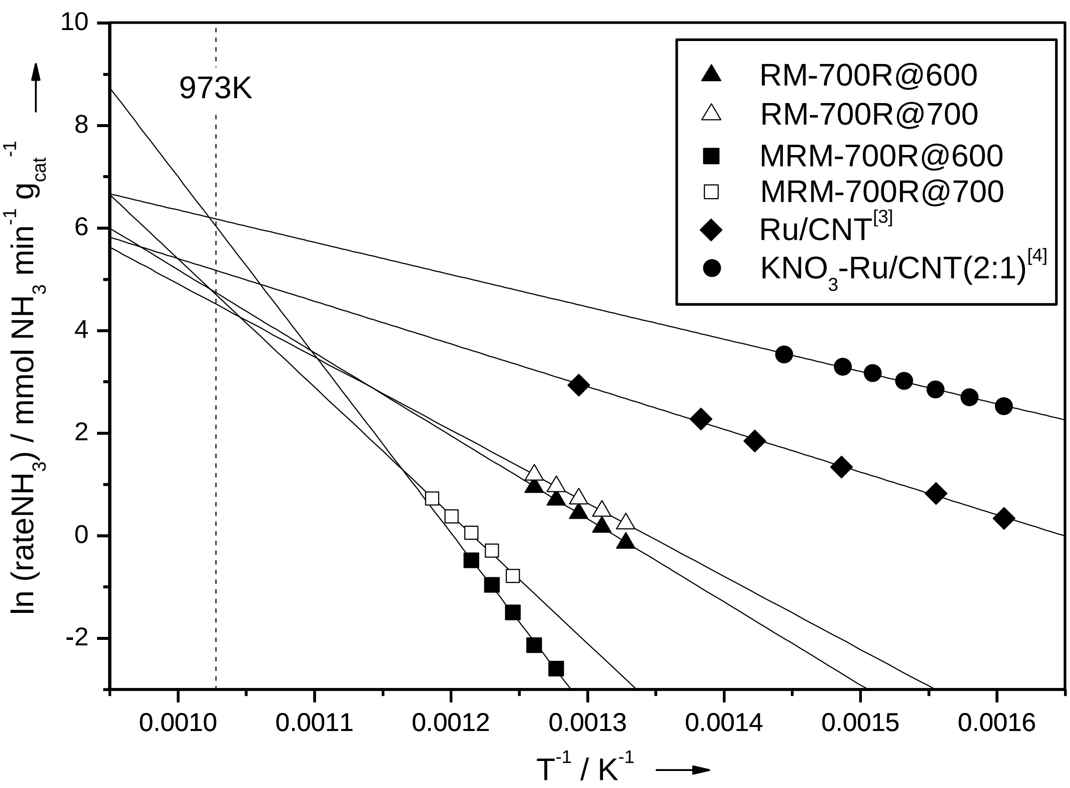

Figure S17: Activity comparison by Arrhenius plots in comparison with Ru/CNT[3], and KNO_3-Ru/CNT(2:1)[4]. It is noted that these values were obtained under differential conversion conditions, thus estimation to higher temperature is only valid under similar low NH_3 conversions.

In Figure S17 the activity of RM and MRM-700R@700 is compared with that of the best performing Ru-based catalysts, Ru/CNT,[3] and KNO_3 modified Ru/CNT catalyst[4] with a K/Ru atomic ratio of 2. We note that this graph provides a rough comparison between the literature data on best performing Ru-based catalysts and MRM-700R@700. The space velocity of MRM-700R@700 for obtaining the Arrhenius plot at differential conversions was 57 775 cm^3 NH_3 h^{-1} g_{cat}^{-1}. Space velocity for Arrhenius plot measurement of Ru/CNT and KNO_3-Ru/CNT(2:1) catalysts were 30 000 and 60 000 cm^3 NH_3 h^{-1} g_{cat}^{-1}, respectively. The dashed

line is showing the location corresponding to the reverse of 700 °C (1/973 K). This rough estimation illustrates that MRM-700R@700 provides on par performance with the Ru-based catalyst at 700 °C. This difference in performance might be because of the higher apparent activation energy observed on MRM-700R@700, which provides higher performance at high temperature. It is noted that this estimation is only valid for differential conversion conditions, at which the Arrhenius plots were obtained.

Additional SEM images of MRM-700@600 and FE-SEM images of MRM-700R@700

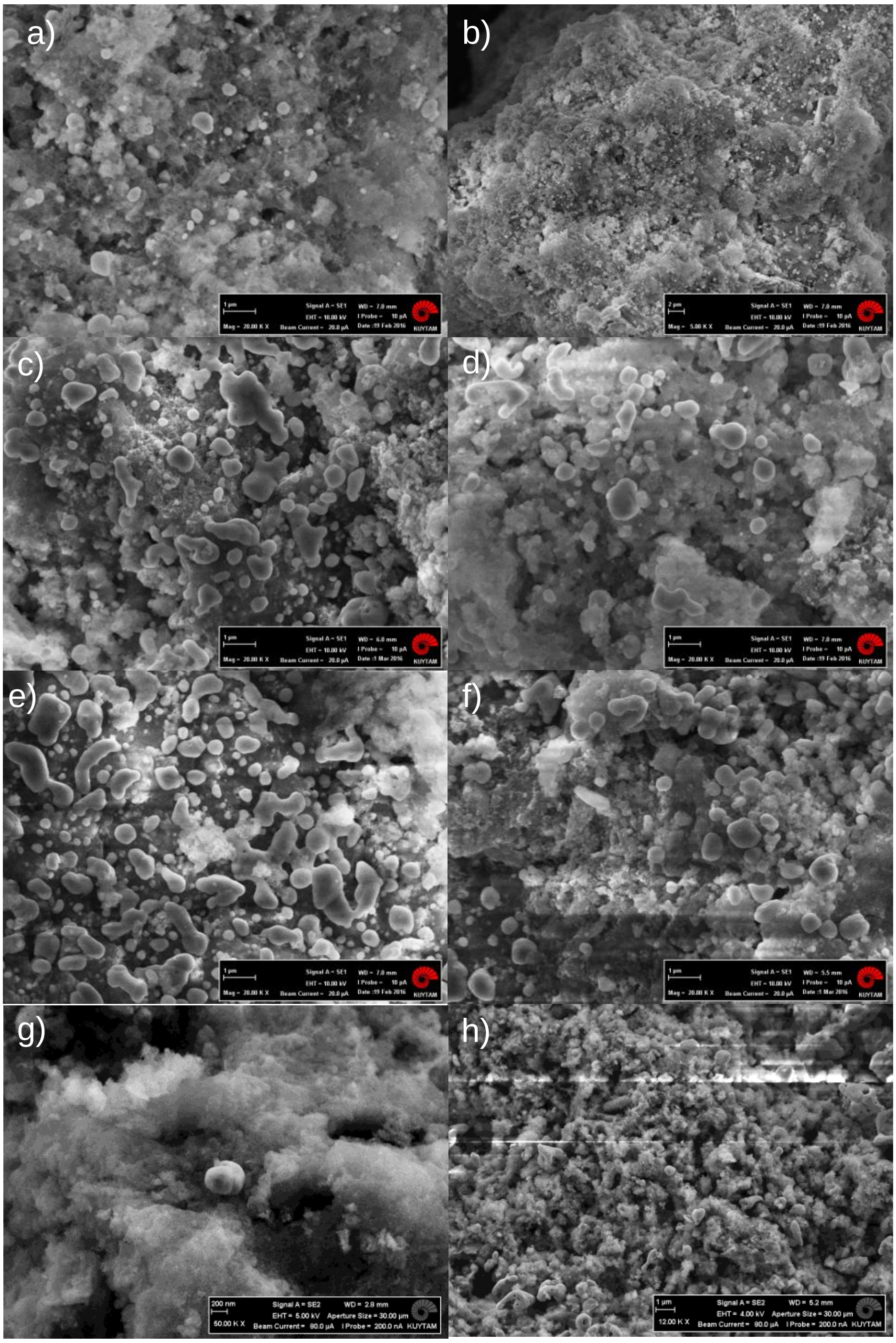

Figure S18: Additional SEM images of MRM-700R@600 at magnifications of 5 000× (in b), 20 000× (in a,c,d,e, and f) and additional FE-SEM images of MRM-700R@700 at magnifications of 50 000× (in g), 12 000× (in h) showing the dispersed iron nitride particles in different locations. The KUYTAM logo in images is published with permission from KUYTAM.

Induction Period for MRM-700R and RM-700R

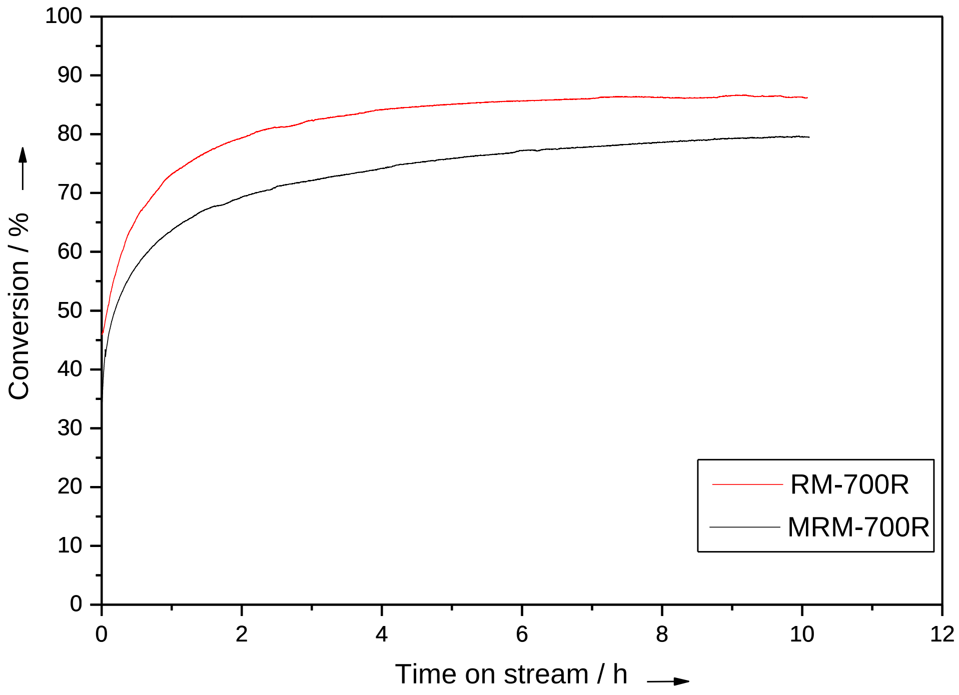

Figure S19: Induction period of RM-700R (in red) and MRM-700R (in black) at a constant space velocity of GHSV of 240 000 cm^3 NH_3 h^{-1} g_{cat}^{-1} at 700°C. Initial point, t = 0 h, indicates the time at which the ammonia flow was started.

According to Figure S19 the induction period is completed after approximately 10 h at 700 °C for RM-700R and MRM-700R. The resulting samples obtained at the end of this induction period were denoted as RM-700R@700 and MRM-700R@700, respectively. After the completion of this period, long-term H_2 production measurements were performed.

Chemical Compositon and Textural Properties

Table S1. Chemical composition of RM, RM-700R, MRM and MRM-700R.

	Composition of chemicals (wt.%)											
Sample	Fe_2O_3	Fe	Al_2O_3	Si_2O	Na_2O	TiO_2	CaO	K_2O	SO_3	ZrO_2	CeO_2	Cl
RM	37.2	-	14.0	11.3	9.3	5.9	1.1	0.30	0.2	0.2	0.1	0.02
RM-700R	-	29.7	16.6	13.5	11.0	6.5	1.3	0.34	0.2	0.2	0.1	0.03
MRM	39.8	-	12.9	10.2	0.2	6.2	0.2	0.02	0.2	0.2	0.1	5.5
MRM-700R	-	35.4	17.8	17.2	0.4	8.0	0.2	0.02	0.1	0.2	0.2	0.3

Table S2. Surface area, pore volume, pore size and N_2 adsorption capacity of RM and MRM at various pressures.

Sample	Surface area (m^2 g^{-1})	Pore volume (cm^3 g^{-1})	Pore size (nm)	Average nanoparticle size (nm)	Excess N_2 Adsorbed at 5 bar (cc(STP)/g)	Excess N_2 Adsorbed at 20 bar (cc(STP)/g)	Excess N_2 Adsorbed at 35 bar (cc(STP)/g)
RM	17.9	0.035	11.1	335.6	0.15	0.28	0.54
MRM	232.4	0.17	5.3	25.8	1.85	6.17	9.78

Comparison of activity with literature focusing on non-noble metal catalysts

Table S3. Comparison of activity with literature

Catalyst	Temperature / °C	Conversion / %	GHSV / $cm^3\ h^{-1}\ g_{cat}^{-1}$	Hydrogen production rate / $mmol\ min^{-1}\ g_{cat}^{-1}$	Reference
RM-700R@500	500	5.1	42 250	2.3	This study
RM-700R@700	500	6.8	44 118	3.1	This study
RM-700R@700	700	99.8	72 000	73.6	This study
RM-700R@700	700	86.2[a]	240 000	212.0	This study
MRM-700R@500	500	0.1	60 000	0.05	This study
MRM-700R@700	500	0.1	40 000	0.03	This study
MRM-700R@700	700	97.4	72 000	71.9	This study
MRM-700R@700	700	93.2	120 000	114.5	This study
MRM-700R@700	700	79.6	240 000	195.7	This study
C-Fe-2.33-400	700	90[a]	120 000	110.4	[3]
C-Fe-2.33-400	700	100[a]	60 000	61.3	[3]
Fe^{III}-Pc-800A-1	700	74.8	60 000	46.1	[4]
Fe-CNFs/CMFs-5	650	100	2 700	2.8	[5]
Fe-CNFs/mica	600	98.9	6 500	6.6	[6]
Fe_3O_4@CeO_2	600	89	24 000	21.8	[7]
α-Fe_2O_3-50@pSiO_2	750	80	120 000	98.1	[8]
Fe@meso-SiO_2-Cs (0.2)	650	100	30 000	33.5	[9]

90FeAl	600	100	18 000	18.3	[10]
K-Ni/MCM-41(TIE)	700	100	30 000	33.5	[11]
Nano-Ni@SiO_2(Si/Ni=0.4)	650	98.6	30 000	33.0	[12]
Ni/LA_2O_3 (40%Ni)	550	78.9	6 000	4.9	[13]
La-modified Ni/Al_2O_3 calcined at 500°C	600	100	6 000	6.2	[14]
La-modified Ni/Al_2O_3 calcined at 400°C reduced at 600°C	700	100	10 000	10.3	[14]
Co/MWCNTs	500	60	6000	3.7	[15]

[a] not stable, deactivates

References

1. Costa, R. C. C. *et al.* Controlled reduction of red mud waste to produce active systems for environmental applications: Heterogeneous Fenton reaction and reduction of Cr(VI). *Chemosphere* **78**, 1116-1120 (2010).
2. Lin, H. Y., Chen, Y. W. & Li, C. The mechanism of reduction of iron oxide by hydrogen. *Thermochim. Acta* **400**, 61-67 (2003).
3. Lu, A. H. *et al.* Spatially and size selective synthesis of Fe-based nanoparticles on ordered mesoporous supports as highly active and stable catalysts for ammonia decomposition. *J. Am. Chem. Soc.* **132**, 14152-14162 (2010).
4. Tüysüz, H., Schüth, F., Zhi, L., Müllen, K. & Comotti, M. Ammonia decomposition over iron phthalocyanine-based materials. *ChemCatChem* **7**, 1453-1459 (2015).
5. Ji, J. *et al.* Fe particles on the tops of carbon nanofibers immobilized on structured carbon microfibers for ammonia decomposition. *Catal. Today* **216**, 254-260 (2013).
6. Duan, X. *et al.* Tuning the size and shape of Fe nanoparticles on carbon nanofibers for catalytic ammonia decomposition. *Applied Catalysis B: Environmental* **101**, 189-196 (2011).
7. Cui, H. Z. *et al.* Iron-based composite nanostructure catalysts used to produce COx-free hydrogen from ammonia. *Science Bulletin*, 1-7 (2016).
8. Feyen, M. *et al.* High-temperature stable, iron-based core-shell catalysts for ammonia decomposition. *Chemistry - A European Journal* **17**, 598-605 (2011).
9. Li, Y. *et al.* Cs-modified iron nanoparticles encapsulated in microporous and mesoporous SiO2 for COx-free H2 production via ammonia decomposition. *Catal. Today* **160**, 79-86 (2011).

10. Gu, Y. Q. *et al.* Transition metal nanoparticles dispersed in an alumina matrix as active and stable catalysts for CO_2 free hydrogen production from ammonia. *Journal of Materials Chemistry A* **3**, 17172-17180 (2015).
11. Li, X. K., Ji, W. J., Zhao, J., Wang, S. J. & Au, C. T. Ammonia decomposition over Ru and Ni catalysts supported on fumed SiO_2, MCM-41, and SBA-15. *J. Catal.* **236**, 181-189 (2005).
12. Yao, L. *et al.* Core-shell structured nickel and ruthenium nanoparticles: Very active and stable catalysts for the generation of COx-free hydrogen via ammonia decomposition. *Catal. Today* **164**, 112-118 (2011).
13. Muroyama, H., Saburi, C., Matsui, T. & Eguchi, K. Ammonia decomposition over Ni/La 2O 3 catalyst for on-site generation of hydrogen. *Applied Catalysis A: General* **443-444**, 119-124 (2012).
14. Okura, K., Okanishi, T., Muroyama, H., Matsui, T. & Eguchi, K. Promotion effect of rare-earth elements on the catalytic decomposition of ammonia over Ni/Al_2O_3 catalyst. *Applied Catalysis A: General* **505**, 77-85 (2015).
15. Zhang, H. *et al.* Tuning catalytic performances of cobalt catalysts for clean hydrogen generation via variation of the type of carbon support and catalyst post-treatment temperature. *Int. J. Hydrogen Energy* **39**, 17573-17582 (2014).
